# Systematic Screening of the Chemical Constituents of Lanqin Oral Liquid by Ultra-High-Performance Liquid Chromatography Combined with Fourier Transform Ion Cyclotron Resonance Mass Spectrometry

**DOI:** 10.3390/molecules28207053

**Published:** 2023-10-12

**Authors:** Ting Liu, Shu Lin

**Affiliations:** School of Pharmacy, Shenyang Medical College, Shenyang 110034, China; linshu@symc.edu.cn

**Keywords:** Lanqin oral liquid, UHPLC-FT-ICR-MS, chemical constituents

## Abstract

A rapid and sensitive method that combined ultra-high-performance liquid chromatography combined with Fourier transform ion cyclotron resonance mass spectrometry (UHPLC-FT-ICR-MS) was used to identify the chemical constituents in Lanqin oral liquid. On the basis of UHPLC-FT-ICR-MS analysis, systematic characterization of the chemical profile of Lanqin oral liquid was carried out, and a total of 441 compounds were identified or tentatively characterized including alkaloids, flavonoids, terpenoids, organic acids, phenylpropanoids, and other types. The results provide a reference for improving quality control, contribute to establishing higher quality standards, provide a scientific basis for further research on the pharmacodynamic material basis, and help illustrate the relationship between the complicated constituents and therapeutic effects of Lanqin oral liquid.

## 1. Introduction

Lanqin oral liquid is the classic traditional Chinese medicine antipyretic prescription. Lanqin oral liquid has the functions of clearing heat, detoxification, liyan, and detumescence effects. The recipe is composed of five valuable herbal medicines, including banlangen-Isatidis Radix, guangqin-Scutellariae Radix, zhizi-Gardeniae Fructus, pangdahai-Sterculiae Lychnophorae Semen, and huangbai-Phellodendri Chinensis Cortex, and has been widely employed in treatments as an antipyretic and anti-inflammatory preparation. Radix Scutellariae possesses anti-inflammatory and antipyretic activities [1,2]. Isatidis Radix has antibacterial, antiviral, antioxidant, anti-inflammatory, and immunomodulatory activities [3]. Gardeniae Fructus has hepatoprotective and choleretic effects and anti-inflammatory, antioxidant, neuroprotective, antidiabetic, antiapoptotic, and antitumor activities [4]. Sterculiae Lychnophorae Semen has anti-inflammatory, analgetic, antibacterial, antiviral, and immunomodulatory activities [5]. Phellodendri Chinensis Cortex has antimicrobic, antitumor, antioxidant, and anti-inflammatory activities [6].

Traditional Chinese medicine (TCM) prescriptions collectively exert therapeutic effects and display a synergistic action via the multicomponents-to-multitargets mechanism guided by the compatibility rules of TCM theory. Given the highly complicated and diverse components in these systems, it is inevitably difficult to identify compounds because these ingredients have rich structural types, wide polarity ranges, and significant differences in content; this always makes the separation and identification challenging and, accordingly, creates difficulties in quality control and the elucidation of pharmacodynamic substances, which hinders modernization. Hence, to ensure the safety and effectiveness of TCM, it is unquestionable that systematic and comprehensive chemical characterization using analytical instruments with high resolution, sensitivity, and accuracy are needed. Recently, the chemical compositions in TCM are increasingly being studied systematically through the use of high-performance liquid chromatography (UHPLC) combined with high-resolution mass spectrometry (HRMS) [7,8]. The overall separation of complex compounds can be efficiently carried out by UPLC; then, these compounds can be accurately characterized and identified by HRMS. A total of 170 and 175 compounds in Lanqin oral liquid were identified or putatively characterized in the previous literature [9,10]. Fourier transform ion cyclotron resonance mass spectrometry (FT-ICR-MS) has been widely used in the identification and characterization of chemical constituents in TCM for high mass accuracy, good sensitivity, high resolution, and rich data [11,12]. A combination of UPLC separation with an FT-ICR-MS system (UPLC-FT-ICR-MS) is suitable for screening and identifying chemical components in complex matrices in terms of the advantages of rapid analysis, high mass accuracy, high resolution, and high detection sensitivity [13,14].

In this study, a rapid, sensitive, and systematic chemical identification and characterization method with UHPLC-FT-ICR-MS was applied to identify and characterize the chemical components of Lanqin oral liquid. A total of 441 compounds including alkaloids, flavonoids, terpenoids, organic acids, phenylpropanoids, and other types were detected, and their structures were tentatively identified based on the retention time, accurate molecular weight measurement, and characteristic mass fragment information with correlative reference standards or reference data. Results of this investigation lay a foundation for the quality control of Lanqin oral liquid; provide a reference and promising experimental data support for further study of the relationship between the effective substances and their pharmacology; illustrate the relationship between the complicated constituents and the therapeutic effects; and, thus, promote the development of Lanqin oral liquid.

## 2. Results and Discussion

### 2.1. Chemical Profiling of Lanqin Oral Liquid

**Figure 1:**
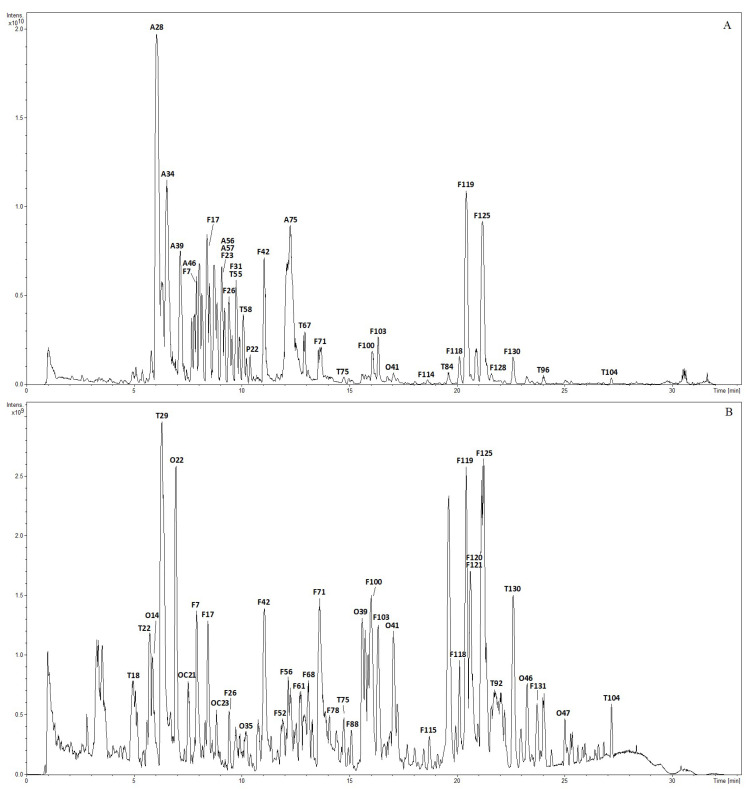
UHPLC-FT-ICR-MS base peak intensity chromatogram in positive (**A**) and negative (**B**) modes of Lanqin oral liquid.

As shown in Figure 1, the representative base peak intensity chromatograms (BPCs) in the positive and negative modes showed the whole profiling of chemical constituents in Lanqin oral liquid. Compounds were identified based on the extracted ion chromatograms, retention time, accurate precursor mass measurements, and diagnostic fragments data. The isomers of compounds with the same molecular formula but different chemical structures were distinguished by characteristic MS/MS fragments and retention time compared with the reference standards and the literature data. In total, 441 chemical constituents, including 134 flavonoids, 88 alkaloids, 108 terpenoids, 51 organic acids, 29 phenylpropanoids, and 31 other compounds in Lanqin oral liquid were unambiguously identified when reference standards were available or tentatively characterized when reference standards were not available by analyzing their MS/MS fragmentation patterns and retention behaviors in comparison with reference standards and the literature data [7,8,9,10,14,15,16,17,18,19,20,21,22,23,24,25,26,27]. The details of the identified compounds are summarized in Table 1, Table 2, Table 3, Table 4 and Table 5.

#### 2.1.1. Characterization and Identification of Flavonoids

**Figure 2:**
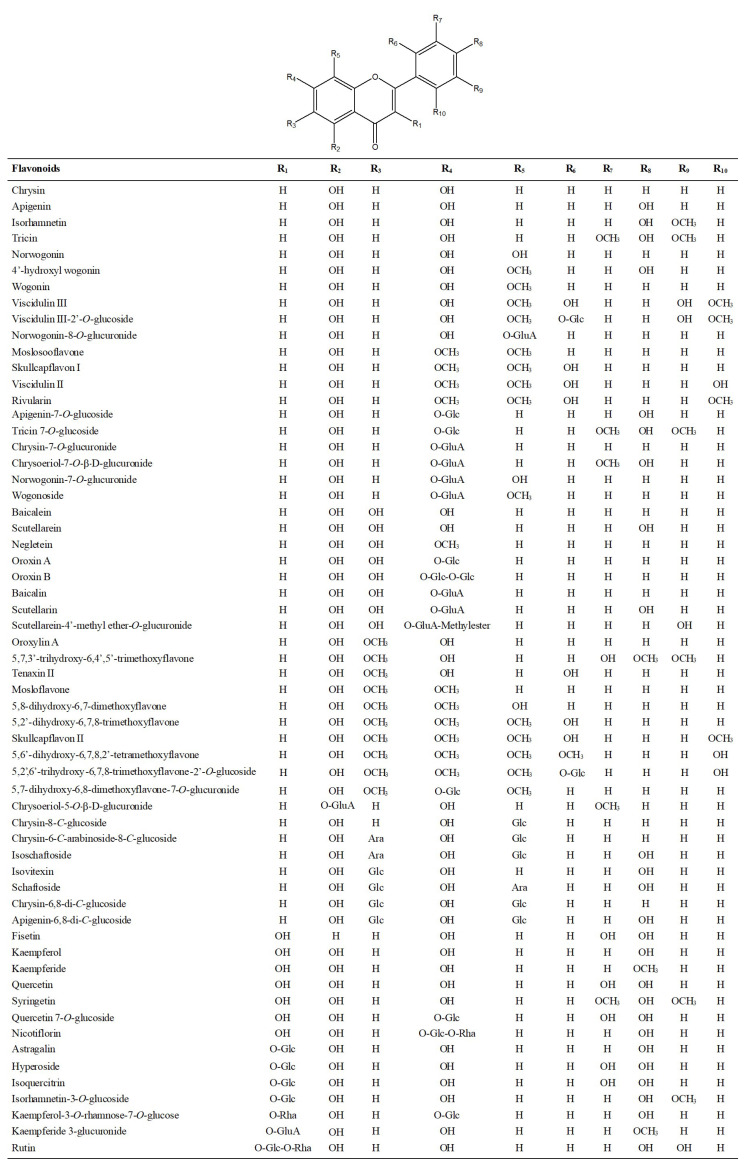
Structures of major flavonoid compounds identified in Lanqin oral liquid. Glu A: glucuronic acid; Glc: glucose; Rha: rhamnose.

Flavonoid compounds are natural compounds containing the basic skeleton C6-C3-C6. A total of 134 flavonoids were tentatively identified in Lanqin oral liquid. The chemical structures of major flavonoid compounds are shown in Figure 2.

**Figure 3:**
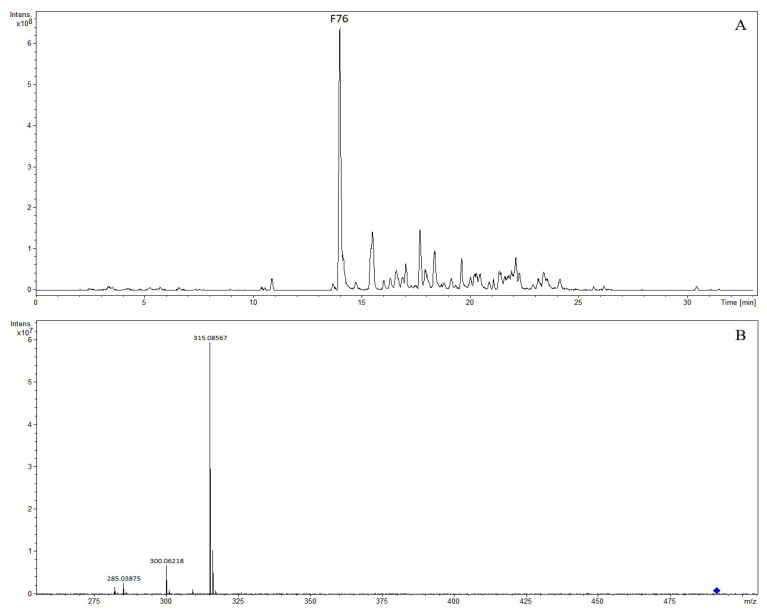
The extracted ion chromatogram (**A**) and MS/MS mass spectrum (**B**) of 5,7-dihydroxy-6,8-dimethoxyflavone-7-*O*-glucuronide in positive mode.

The precursor ion [M-H]^−^ of compound F12 was at *m*/*z* 461.07229 and was observed at 8.22 min. The molecular formula was C_21_H_18_O_12_, as predicted by the precursor ion. The fragment ion at *m*/*z* 285.04039 was observed, indicating the elimination of a glucuronosyl unit in its structure. The compound was identified as scutellarin [9]. Compound F31 was found at 9.72 min, and the molecular formula was C_24_H_26_O_13_, as established by the [M-H]^−^ peak at *m*/*z* 521.12952. The characteristic fragments were *m*/*z* 359.07727, *m*/*z* 344.05343, and *m*/*z* 329.02992, corresponding to an excimer ion [M + H]^+^ successively removing a fragment ion of glucose, one and two methyl groups. It was tentatively characterized as 5,2′,6′-trihydroxy-6,7,8-trimethoxyflavone-2′-*O*-glucoside [7]. Compound F76 with a retention time of 13.97 min generated the precursor ion of [M + H]^+^ at *m*/*z* 491.11836 in the negative ion mode, and the molecular formula was predicted as C_23_H_22_O_12_. The characteristic fragment were *m*/*z* 315.08567, *m*/*z* 300.06218, and *m*/*z* 285.03875, corresponding to an excimer ion [M + H]^+^ successively removing a fragment ion of glucose, one and two methyl groups. It was tentatively characterized as 5,7-dihydroxy-6,8-dimethoxyflavone-7-*O*-glucuronide [7]. The extracted ion chromatogram and MS/MS mass spectrum of 5,7-dihydroxy-6,8-dimethoxyflavone-7-*O*-glucuronide are shown in Figure 3. The cleavage at the glycosidic linkages was the main fragmentation pattern of *O*-glycoside.

The precursor ions [M + H]^+^ of compound F26 and compound F46 were at *m*/*z* 417.11859 and *m*/*z* 417.11836 and were observed at 9.40 and 11.24 min; the molecular formula was C_21_H_20_O_9_, as predicted by the precursor ion. In the MS/MS spectrum, the characteristic fragment ions of compound F26 at *m*/*z* 399.10614, *m*/*z* 381.09575, *m*/*z* 363.08537, *m*/*z* 351.08537, *m*/*z* 339.08545, *m*/*z* 335.09070, *m*/*z* 327.08588, *m*/*z* 321.07497, *m*/*z* 307.09563, *m*/*z* 297.07507, *m*/*z* 279.06454, and *m*/*z* 267.06457 were detected, which corresponded to [M + H-H_2_O]^+^, [M + H-2H_2_O]^+^, [M + H-3H_2_O]^+^, [M + H-CH_2_O-2H_2_O]^+^, [M + H-C_2_H_4_O_2_-H_2_O]^+^, [M + H-3H_2_O-CO]^+^, [M + H-C_3_H_6_O_3_]^+^, [M + H-C_2_H_4_O_2_-2H_2_O]^+^, [M + H-3H_2_O-2CO]^+^, [M + H-C_4_H_8_O_4_]^+^, [M + H-C_4_H_8_O_4_-H_2_O]^+^, and [M + H-C_5_H_10_O_5_]^+^, respectively. The characteristic fragment ions of compound F46 at *m*/*z* 381.09610, *m*/*z* 363.08528, *m*/*z* 351.08569, *m*/*z* 345.07486, *m*/*z* 335.09070, *m*/*z* 333.07491, *m*/*z* 321.07497, *m*/*z* 307.09570, *m*/*z* 297.07508, *m*/*z* 293.08047, *m*/*z* 279.06454, and *m*/*z* 267.06455 were detected, which corresponded to [M + H-2H_2_O]^+^, [M + H-3H_2_O]^+^, [M + H-CH_2_O-2H_2_O]^+^, [M + H-4H_2_O]^+^, [M + H-3H_2_O-CO]^+^, [M + H-CH_2_O-3H_2_O]^+^, [M + H-C_2_H_4_O_2_-2H_2_O]^+^, [M + H-3H_2_O-2CO]^+^, [M + H-C_4_H_8_O_4_]^+^, [M+H-C_2_H_4_O_2_-2H_2_O-CO]^+^, [M + H-C_4_H_8_O_4_-H_2_O]^+^, and [M + H-C_5_H_10_O_5_]^+^, respectively. Compound F26 and compound F46 were tentatively characterized as chrysin 8-*C*-glucoside and chrysin 6-*C*-glucoside [15]. The ions produced by losing CH_2_O, C_2_H_4_O_2_, C_3_H_6_O_3_, C_4_H_8_O_4_, C_5_H_10_O_5_, and water molecules from the precursor ions were the main fragmentation patterns of *C*-glycoside.

**Figure 4:**
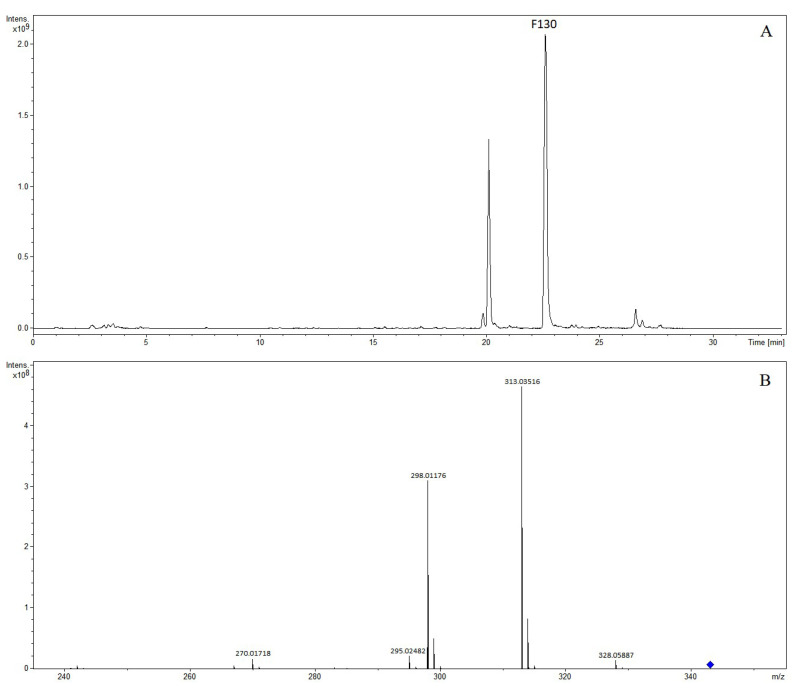
The extracted ion chromatogram (**A**) and MS/MS mass spectrum (**B**) of 5,2′-dihydroxy-6,7,8-trimethoxyflavone in negative mode.

Compounds F118 and F130 with a retention time of 20.12 min and 22.60 min generated the precursor ion of [M-H]^−^ at *m*/*z* 343.08212 and *m*/*z* 343.08199 in the negative ion mode, and the molecular formula was predicted as C_18_H_15_O_7_. In the MS/MS experiments of compound F118, fragment ions at *m*/*z* 328.05879, *m*/*z* 313.03510, *m*/*z* 298.01173, *m*/*z* 285.04033, and *m*/*z* 270.01684 corresponded to [M-H-CH_3_]^−^, [M-H-2CH_3_]^−^, [M-H-3CH_3_]^−^, [M-H-2CH_3_-CO]^−^, and [M-H-3CH_3_-CO]^−^. In the MS/MS experiments of compound F130, fragment ions at *m*/*z* 328.05887, *m*/*z* 313.03516, *m*/*z* 298.01176, *m*/*z* 295.02482, and *m*/*z* 270.01718 corresponded to [M-H-CH_3_]^−^, [M-H-2CH_3_]^−^, [M-H-3CH_3_]^−^, [M-H-2CH_3_-H_2_O]^−^, and [M-H-3CH_3_-CO]^−^. Compounds F118 and F130 were tentatively characterized as rivularin and 5,2′-dihydroxy-6,7,8-trimethoxyflavone [8]. The extracted ion chromatogram and MS/MS mass spectrum of 5,2′-dihydroxy-6,7,8-trimethoxyflavone are shown in Figure 4.

#### 2.1.2. Characterization and Identification of Alkaloids

**Figure 5:**
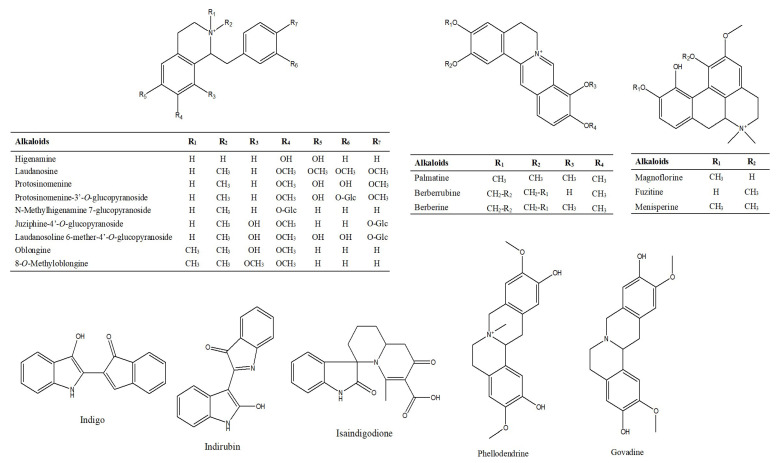
Structures of major alkaloid compounds identified in Lanqin oral liquid.

Alkaloids are a class of basic organic compounds containing nitrogen that exist in nature. A total of 88 alkaloids were tentatively identified in Lanqin oral liquid, mainly containing tetrahydroperoberberines such as phellodendrine, aporphines such as magnoflorine, benzylisoquinoline such as *N*-methylhigenamine 7-glucopyranoside, protoberberines such as palmatine, and other types such as indirubin. The chemical structures of the major alkaloid compounds are shown in Figure 5.

**Figure 6:**
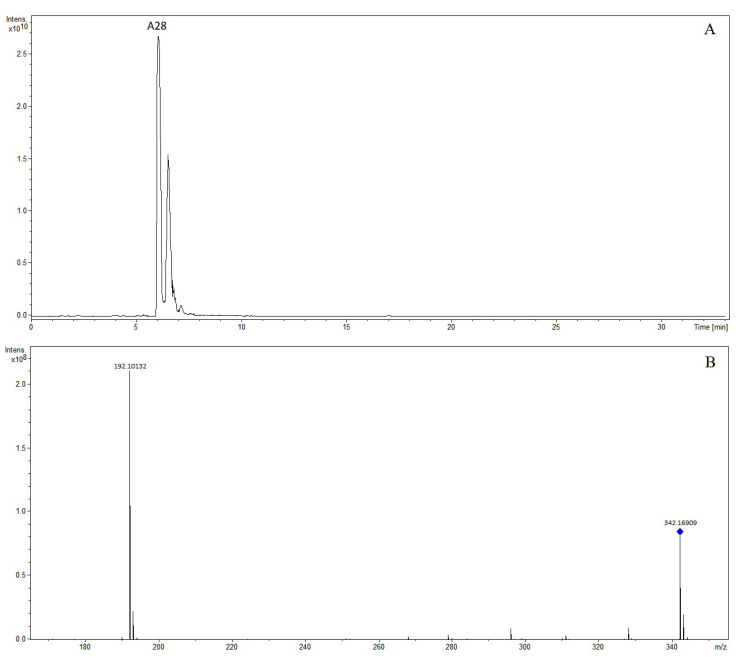
The extracted ion chromatogram (**A**) and MS/MS mass spectrum (**B**) of phellodendrine in positive mode.

The precursor ion of compound A28 at *m*/*z* 342.16909 was observed at 6.05 min. We speculated that the molecular formula was C_20_H_24_NO_4_. In the MS/MS spectrum, the characteristic fragment ion produced was at *m*/*z* 192.10132, corresponding to fragment ion [M-C_9_H_10_O_2_]^+^. The compound was tentatively characterized as phellodendrine [9,10]. The extracted ion chromatogram and MS/MS mass spectrum of phellodendrine are shown in Figure 6.

The precursor ion of compound A34 at *m*/*z* 342.16988 was observed at 6.52 min. We speculated that the molecular formula was C_20_H_24_NO_4_. In the MS/MS spectrum, the characteristic fragment ions produced were at *m*/*z* 297.11172, *m*/*z* 282.08828, *m*/*z* 265.08555, and *m*/*z* 237.09072, corresponding to fragment ions [M-NH(CH_3_)_2_]^+^, [M-NH(CH_3_)_2_-CH_3_]^+^, [M-NH(CH_3_)_2_-CH_3_OH]^+^, and [M-NH(CH_3_)_2_-CH_3_OH-CO]^+^. The compound was tentatively characterized as magnoflorine [16].

**Figure 7:**
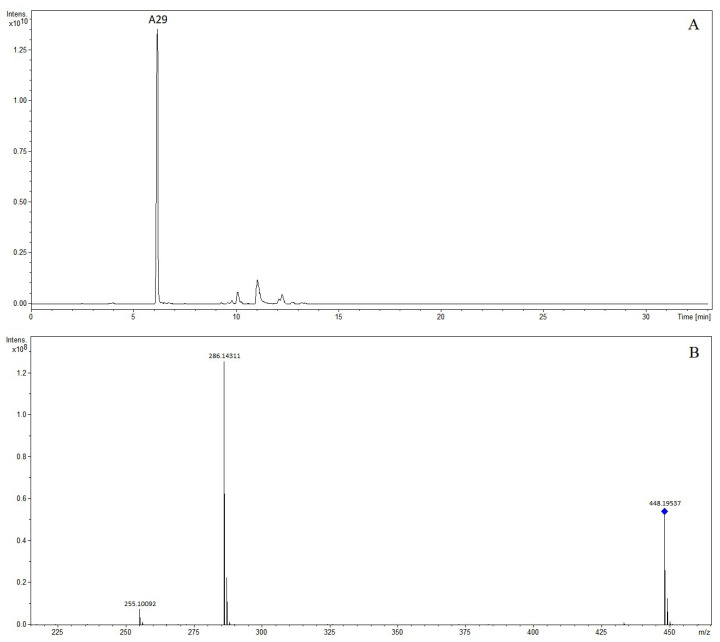
The extracted ion chromatogram (**A**) and MS/MS mass spectrum (**B**) of *N*-methylhigenamine 7-glucopyranoside in positive mode.

The precursor ion of compound A29 at *m*/*z* 448.19537 was observed at 6.15 min. We speculated that the molecular formula was C_23_H_29_NO_8_. In the MS/MS spectrum, the characteristic fragment ions at *m*/*z* 286.14311 and *m*/*z* 255.10092 were detected, which corresponded to [M + H-glucose]^+^ and [M + H-glucose-NH_2_CH_3_]^+^. The compound was tentatively characterized as *N*-methylhigenamine 7-glucopyranoside [10]. The extracted ion chromatogram and MS/MS mass spectrum of *N*-methylhigenamine 7-glucopyranoside are shown in Figure 7.

The precursor ion of compound A74 at *m*/*z* 352.15457 was observed at 11.97 min. We speculated that the molecular formula was C_21_H_22_NO_4_. In the MS/MS spectrum, the characteristic fragment ions at *m*/*z* 336.12260, *m*/*z* 320.12862, and *m*/*z* 308.12759 were detected, which corresponded to [M-CH_4_]^+^, [M-CH_2_O]^+^, and [M-CH_4_-CO]^+^. The compound was chemically defined as palmatine [9,10].

The precursor ion [M + H]^+^ of compound A86 was at *m*/*z* 263.08145 and was observed at 23.49 min. The molecular formula was C_16_H_11_N_2_O_2_, as predicted by the precursor ion. In the MS/MS spectrum, the characteristic fragment ions at *m*/*z* 245.07024, *m*/*z* 235.08585, and *m*/*z* 219.09110 corresponded to [M + H-H_2_O]^+^, [M+H-CO]^+^, and [M + H-CO_2_]^+^. The compound was chemically defined as indirubin. The precursor ion [M + H]^+^ of compound A88 was at *m*/*z* 263.08146 and was observed at 21.69 min. The MS spectra of the two compounds were highly similar to each other. The compound was chemically defined as indigo [17].

#### 2.1.3. Characterization and Identification of Terpenoids

**Figure 8:**
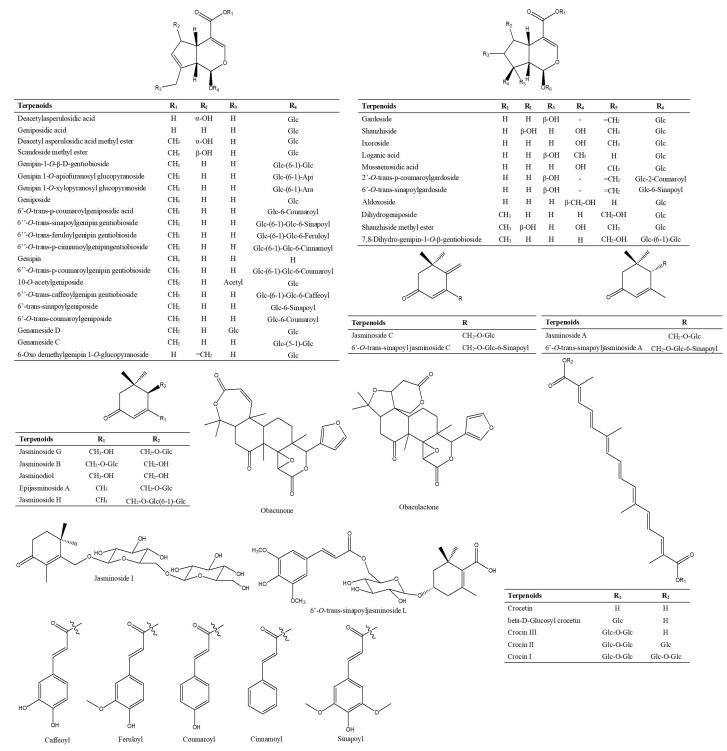
Structures of major terpenoid compounds identified in Lanqin oral liquid.

Terpenoid compounds are compounds and derivatives derived from methylglutaric acid, and their basic structural unit is a prenyl unit (C5 unit). A total of 108 terpenoids were tentatively identified in Lanqin oral liquid, mainly contain iridoids such as geniposidic acid, monoterpenoids such as jasminoside B, diterpenoids such as crocin I, and triterpenoids such as obacunone. The chemical structures of major terpenoid compounds are shown in Figure 8.

**Figure 9:**
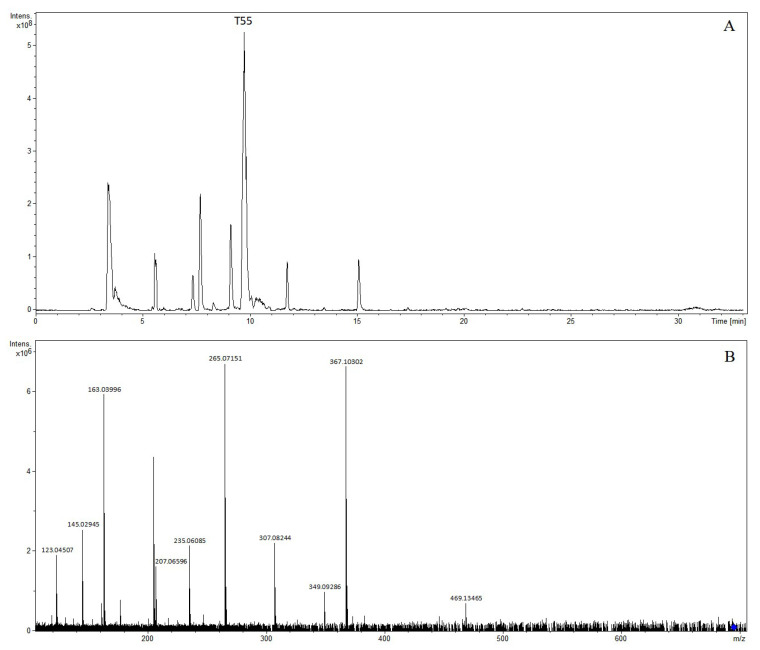
The extracted ion chromatogram (**A**) and MS/MS mass spectrum (**B**) of 6″-*O*-trans-p-coumaroylgenipin gentiobioside in negative mode.

The precursor ion [M-H]^−^ of compound T5 was at *m*/*z* 373.11374 and was observed at 2.34 min, indicating the formation of C_16_H_22_O_10_. The characteristic fragments were *m*/*z* 211.06098, *m*/*z* 193.05035, *m*/*z* 167.07122, *m*/*z* 149.06069, and *m*/*z* 123.04507, corresponding to fragment ions [M-H-Glc]^−^, [M-H-Glc-H_2_O]^−^, [M-H-Glc-CO_2_]^−^, [M-H-Glc-H_2_O-CO_2_]^−^, and [M-H-Glc-H_2_O-CO_2_-C_2_H_2_]^−^. The compound was tentatively characterized as geniposidic acid [9,10,18]. The precursor ion [M-H]^−^ of compound T38 was at *m*/*z* 225.07668 and was observed at 7.36 min, indicating the formation of C_11_H_14_O_5_. Daughter ions at *m*/*z* 147.04498 [M-H-C_2_H_6_O_3_]^−^ were observed in the MS/MS spectrum. The compound was tentatively characterized as genipin [18,19,20]. Compound T22 was found at 5.70 min, and the molecular formula was C_23_H_34_O_15_, as established by the [M + COOH]^−^ peak at *m*/*z* 595.18640 and the [M-H]^−^ peak at *m*/*z* 549.18166. Daughter ions at *m*/*z* 225.07659 [M-H-2C_6_H_10_O_5_]^−^, *m*/*z* 207.06613 [M-H-2C_6_H_10_O_5_-H_2_O]^−^, *m*/*z* 147.04499 [M-H-2C_6_H_10_O_5_-C_2_H_6_O_3_]^−^, *m*/*z* 123.04505 [M-H-2C_6_H_10_O_5_-H_2_O-C_4_H_4_O_2_]^−^, and *m*/*z* 101.02435 [M-H-2C_6_H_10_O_5_-C_7_H_8_O_2_]^−^ were observed in the MS/MS spectrum. Compound T22 was tentatively characterized as genipin-1-*O*-β-D-gentiobioside [10,18,19,20]. The precursor ion [M-H]^−^ of compound T55 was at *m*/*z* 695.21845 and was observed at 9.72 min, indicating the formation of C_32_H_40_O_17_. Daughter ions at *m*/*z* 469.13465 [M-H-C_11_H_14_O_5_]^−^, *m*/*z* 367.10302 [M-H-C_15_H_20_O_8_]^−^, *m*/*z* 349.09286 [M-H-C_15_H_20_O_8_-H_2_O]^−^, *m*/*z* 307.08244 [M-H-C_11_H_14_O_5_-C_6_H_10_O_5_]^−^, *m*/*z* 265.07151 [M-H-C_19_H_26_O_11_]^−^, *m*/*z* 235.06085 [M-H-C_20_H_28_O_12_]^−^, *m*/*z* 207.06596 [M-H-C_21_H_28_O_13_]^−^, *m*/*z* 163.03996 [M-H-C_11_H_12_O_4_-2C_6_H_10_O_5_]^−^, *m*/*z* 145.02945 [M-H-C_11_H_12_O_4_-2C_6_H_10_O_5_-H_2_O]^−^, and *m*/*z* 123.04507 [M-H-C_9_H_6_O_2_-2C_6_H_10_O_5_-H_2_O-C_4_H_4_O_2_]^−^ were observed in the MS/MS spectrum. The compound was tentatively characterized as 6″-*O*-trans-p-coumaroylgenipin gentiobioside [19,20,21]. The extracted ion chromatogram and MS/MS mass spectrum of 6″-*O*-trans-p-coumaroylgenipin gentiobioside are shown in Figure 9.

The precursor ions [M + COOH]^−^ and [M-H]^−^ of compound T18 were at *m*/*z* 391.16044 and *m*/*z* 345.15548 and were observed at 4.96 min, indicating the formation of C_16_H_26_O_8_. The characteristic fragment ions at *m*/*z* 179.05598 [M-H-C_10_H_14_O_2_]^−^, *m*/*z* 165.09202 [M-H-C_6_H_12_O_6_]^−^, and *m*/*z* 161.04543 [M-H-C_10_H_16_O_3_]^−^ were observed in the MS/MS spectrum. The compound was tentatively characterized as jasminoside B [10].

The precursor ion [M-H]^−^ of compound T60 was at *m*/*z* 975.37046 observed at 10.34 min, indicating the formation of C_44_H_63_O_24_. Daughter ions at *m*/*z* 651.26536 [M-H-2glucose]^−^ were observed in the MS/MS spectrum. The compound was tentatively characterized as crocin I [9].

**Figure 10:**
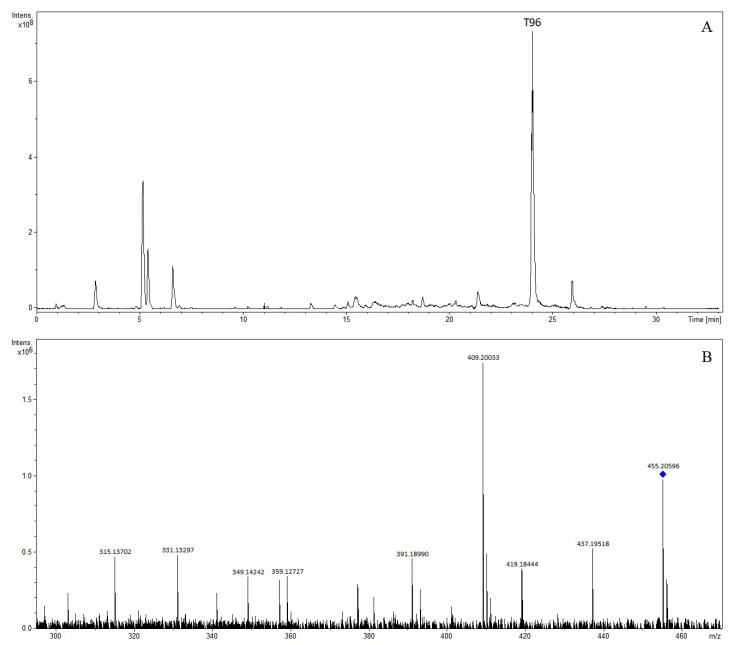
The extracted ion chromatogram (**A**) and MS/MS mass spectrum (**B**) of obacunone in positive mode.

The precursor ion [M + H]^+^ of compound T96 was at *m*/*z* 455.20596 and was observed at 24.02 min, indicating the formation of C_26_H_30_O_7_. Daughter ions at *m*/*z* 437.19518 [M + H-H_2_O]^+^, *m*/*z* 419.18444 [M + H-2H_2_O]^+^, *m*/*z* 409.20033 [M + H-CH_2_O_2_]^+^, *m*/*z* 391.1899 [M + H-CH_2_O_2_-H_2_O]^+^, 359.12727 [M + H-C_3_H_12_O_3_]^+^, 349.14242 [M + H-C_4_H_10_O_3_]^+^, 331.13297 [M + H-C_4_H_12_O_4_]^+^, and 315.13702 [M + H-C_4_H_12_O_5_]^+^ were observed in the MS/MS spectrum. The compound was tentatively characterized as obacunone [22]. The extracted ion chromatogram and MS/MS mass spectrum of obacunone are shown in Figure 10.

#### 2.1.4. Characterization and Identification of Organic Acids

**Figure 11:**
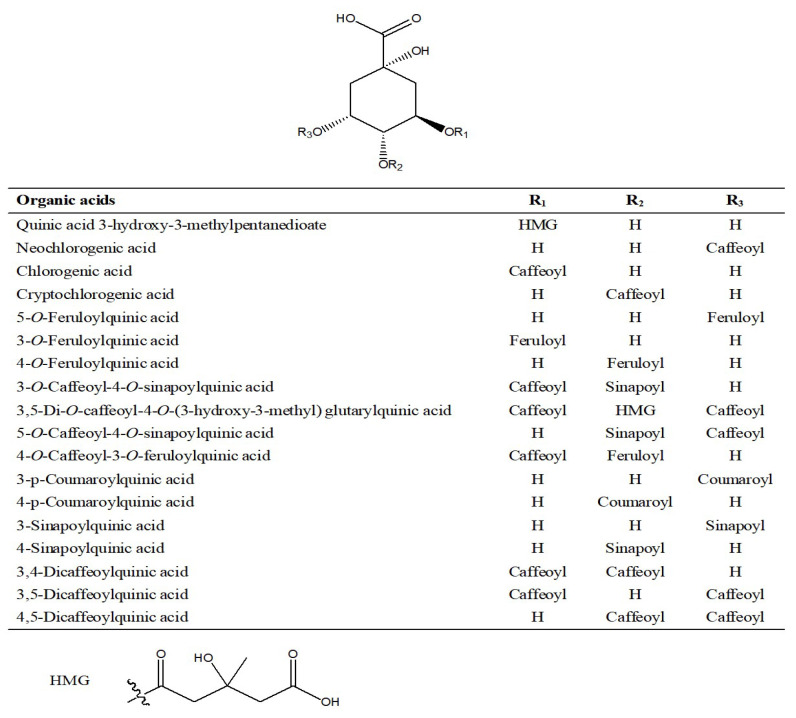
Structures of major organic acid compounds identified in Lanqin oral liquid.

**Figure 12:**
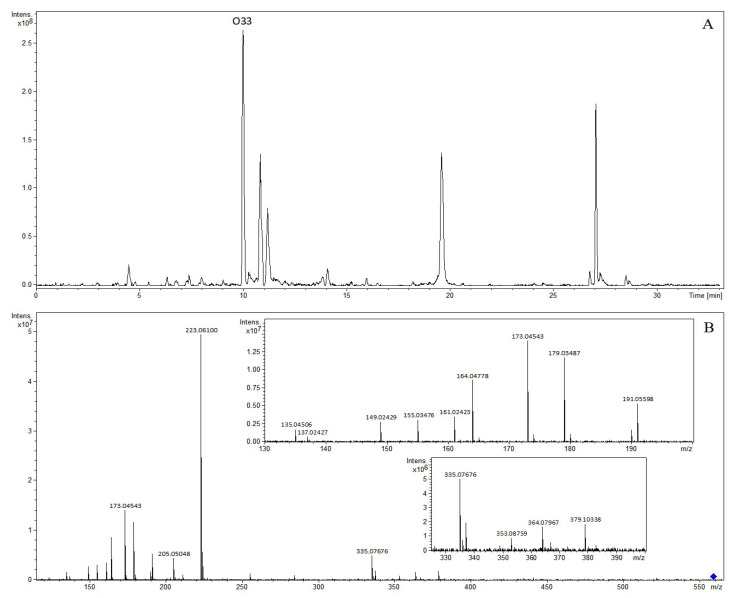
The extracted ion chromatogram (**A**) and MS/MS mass spectrum (**B**) of 3-*O*-caffeoyl-4-*O*-sinapoylquinic acid in negative mode.

Organic acids refer to some organic compounds with acidity, most commonly containing a carboxyl group (–COOH, 44 Da). A total of 51 organic acids were tentatively identified in Lanqin oral liquid. The chemical structures of major organic acid compounds are shown in Figure 11. Compound O1 with a retention time of 0.94 min generated the precursor ion of [M-H]^−^ at *m*/*z* 191.05582 in negative ion mode, and the molecular formula was predicted as C_7_H_12_O_6_. In the MS/MS spectrum, the characteristic fragment ions produced were at *m*/*z* 173.04533 and *m*/*z* 127.03983, corresponding to fragment ions [M-H-H_2_O]^−^ and [M-H-H_2_O-H_2_CO_2_]^−^. It was tentatively characterized as quinic acid [8]. Compound O17 with a retention time of 6.17 min generated the precursor ion of [M-H]^−^ at *m*/*z* 179.03487 in negative ion mode, and the molecular formula was predicted as C_9_H_8_O_4_. In the MS/MS spectrum, the characteristic fragment ion produced was at *m*/*z* 135.04512, corresponding to fragment ion [M-H-CO_2_]^−^. It was identified as caffeic acid [8,18]. The precursor ion [M-H]^−^ of compound O33 was at *m*/*z* 559.14509 and was observed at 9.99 min, indicating the formation of C_27_H_28_O_13_. Daughter ions at *m*/*z* 379.10338 [M-H-C_9_H_8_O_4_]^−^, *m*/*z* 364.07967 [M-H-C_10_H_11_O_4_]^−^, *m*/*z* 353.08759 [M-H-C_11_H_10_O_4_]^−^, *m*/*z* 335.07676 [M-H-C_11_H_12_O_5_]^−^, *m*/*z* 223.06100 [M-H-C_16_H_16_O_8_]^−^, *m*/*z* 205.05048 [M-H-C_16_H_18_O_9_]^−^, *m*/*z* 191.05598 [M-H-C_20_H_16_O_7_]^−^, *m*/*z* 179.03487 [M-H-C_18_H_20_O_9_]^−^, *m*/*z* 173.04543 [M-H-C_20_H_18_O_8_]^−^, *m*/*z* 164.04778 [M-H-C_18_H_19_O_10_]^−^, *m*/*z* 161.02423 [M-H-C_18_H_22_O_10_]^−^, *m*/*z* 155.03476 [M-H-C_20_H_20_O_9_]^−^, *m*/*z* 149.02429 [M-H-C_19_H_22_O_10_]^−^, *m*/*z* 137.02427 [M-H-C_20_H_22_O_10_]^−^, and *m*/*z* 135.04506 [M-H-C_19_H_20_O_11_]^−^ were observed in the MS/MS spectrum. Fragment ions at *m*/*z* 379.10338 and *m*/*z* 364.07967 corresponded to successive losses of caffeoyl and methyl groups. Fragment ions at *m*/*z* 353.08759 and *m*/*z* 335.07680 corresponded to successive losses of a sinapoyl group and H_2_O. Fragment ions at *m*/*z* 223.06100, *m*/*z* 205.05048, and *m*/*z* 164.04778 corresponded to successive losses of caffeoyl + quinic acid, H_2_O, and CO_2_, and also can be regarded as [M-H]^−^, [M-H-H_2_O]^−^, and [M-H-H_2_O-CO_2_]^−^ ions of sinapinic acid. Fragment ions at *m*/*z* 191.05598, *m*/*z* 173.04543, *m*/*z* 155.03476, and *m*/*z* 137.02427 corresponded to successive losses of a caffeoyl + sinapoyl group, H_2_O, H_2_O, and CO_2_. Fragment ions at *m*/*z* 179.03487, *m*/*z* 161.02423, and *m*/*z* 135.04506 corresponded to losses of sinapoyl + quinic acid, H_2_O, and CO_2_, and also can be regarded as [M-H]^−^, [M-H-H_2_O]^−^, and [M-H-H_2_O-CO_2_]^−^ ions of caffeic acid. The compound was tentatively characterized as 3-*O*-caffeoyl-4-*O*-sinapoylquinic acid [10]. The extracted ion chromatogram and MS/MS mass spectrum of 3-*O*-caffeoyl-4-*O*-sinapoylquinic acid are shown in Figure 12.

#### 2.1.5. Characterization and Identification of Phenylpropanoids

**Figure 13:**
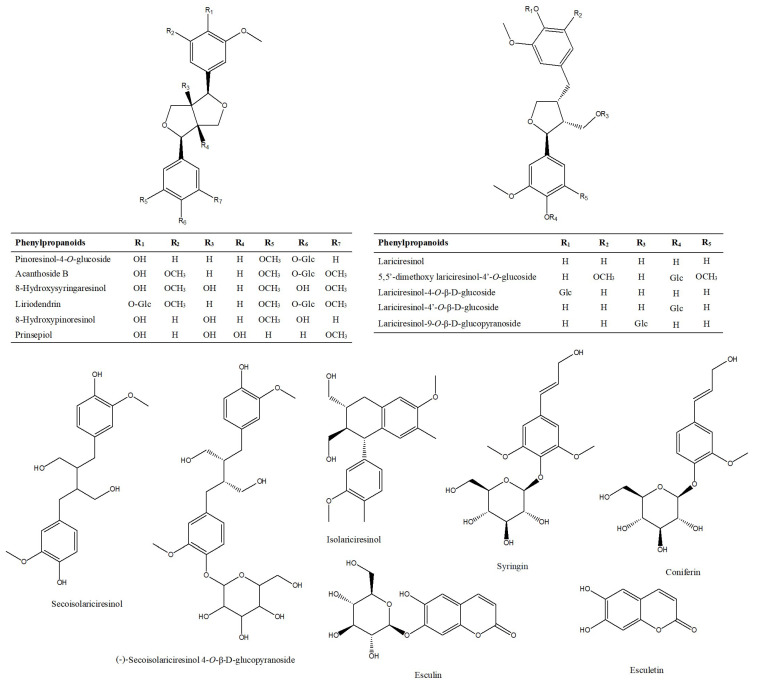
Structures of major phenylpropanoid compounds identified in Lanqin oral liquid.

**Figure 14:**
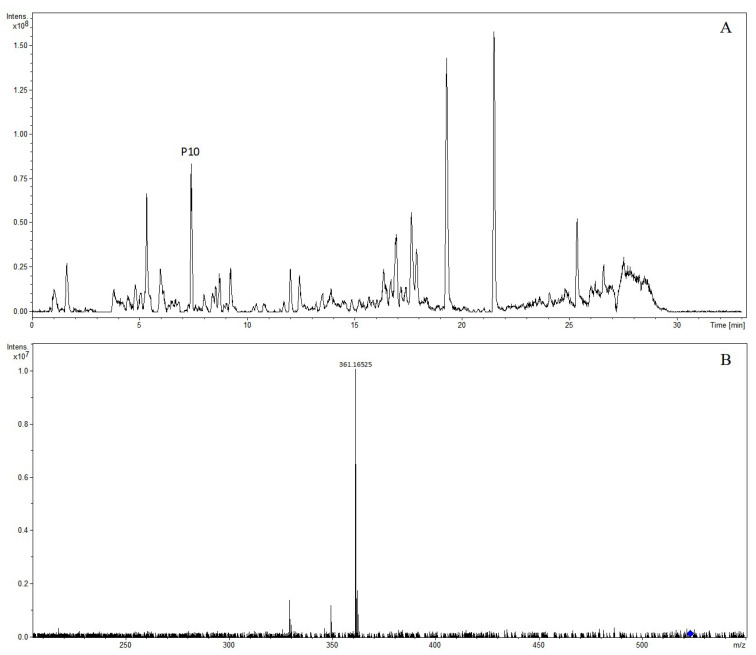
The extracted ion chromatogram (**A**) and MS/MS mass spectrum (**B**) of (−)-secoisolariciresinol 4-*O*-β-D-glucopyranoside in negative mode.

Phenylpropanoid compounds are natural compounds with one or more C6-C3 as the basic skeleton. A total of 29 phenylpropanoids were tentatively identified in Lanqin oral liquid. The chemical structures of major phenylpropanoid compounds are shown in Figure 13. The precursor ion [M + H]^+^ of compound P1 was at *m*/*z* 341.08642 and was observed at 4.40 min. The molecular formula was predicted as C_15_H_16_O_9_. In the MS/MS spectrum, the characteristic fragment ion produced was at *m*/*z* 179.03357 [M + H-glucose]^+^. The compound was tentatively characterized as esculin [23]. The precursor ion [M + COOH]^−^ of compound P2 was at *m*/*z* 387.12932 and was observed at 5.03 min, indicating the formation of C_16_H_22_O_8_. In the MS/MS spectrum, the characteristic ions at *m*/*z* 341.12388 [M-H]^−^ and *m*/*z* 179.07122 [M-H-glucose]^−^ were observed. The compound was tentatively characterized as coniferin [24]. The precursor ion [M-H]^−^ of compound P10 was at *m*/*z* 523.21827 and was observed at 7.40 min. The molecular formula was predicted as C_26_H_36_O_11_. In the MS/MS spectrum, the characteristic fragment ion produced was at *m*/*z* 361.16525, which corresponded to [M-H-C_6_H_10_O_5_]^−^. The compound was tentatively characterized as (−)-secoisolariciresinol 4-*O*-β-D-glucopyranoside [8]. The extracted ion chromatogram and MS/MS mass spectrum of (−)-secoisolariciresinol 4-*O*-β-D-glucopyranoside are shown in Figure 14.

According to the above analysis results, chemical identification of the compounds was performed based on an integrated consideration of accurate MS data, characteristic fragment ions, and retention time using a reference substance or the reference literature. A total of 441 compounds in Lanqin oral liquid were identified in this study. In the previous literature, a total of 170 compounds were identified or putatively characterized by ultra-high-performance liquid chromatography coupled with ion mobility-quadrupole time-of-flight mass spectrometry [9], and 175 compounds were identified or putatively characterized by ultra-high-performance liquid chromatography coupled with quadrupole time-of-flight mass spectrometry in Lanqin oral liquid [10], while 441 compounds were identified or tentatively characterized in Lanqin oral liquid by UHPLC-FT-ICR-MS/MS. It was found that the applied UHPLC-FT-ICR-MS/MS method with high mass accuracy, good sensitivity, and high resolution was more sensitive for detecting and identifying the chemical compounds in Lanqin oral liquid.

Compounds including flavonoids, alkaloids, terpenoids, organic acids, and phenylpropanoids were identified in Lanqin oral liquid. The content of compounds, such as baicalin, wogonoside, baicalein, wogonin, phellodendrine, magnoflorine, berberine, palmatine, indirubin, genipin-1-*O*-β-D-gentiobioside, geniposide, obaculactone, obacunone, and chlorogenic acid, were determined in Lanqin oral liquid [28]. Based on the combined content and pharmacological activity studies of the compounds, these chemical constituents with high content and pharmacological activity could be the pharmacodynamic substances that play an important role in vivo. Flavonoids, for example, baicalein, baicalin, wogonin, wogonoside, oroxylin A, and scutellarin, show anti-inflammatory activity [29,30], and baicalin also shows an antipyretic effect [31]. Alkaloids, for example, phellodendrine and berberine, show anti-inflammatory activity [32,33], and indigo and indirubin show antioxidant activity [34]. Terpenoids, for example, geniposidic acid, protects LPS-induced ALI through the TLR4/MyD88 signaling pathway [35], and geniposide, genipin, and crocin-I show anti-inflammatory activity [36,37,38]. Obacunone shows antioxidant and anti-inflammatory activities [39]. Organic acids, for example, caffeic acid and chlorogenic acid, show antioxidant, antibacterial, and anti-inflammatory activities [40,41]. Phenylpropanoids, for example, esculin, shows anti-inflammatory and antioxidative stress effects [42]; pinoresinol-4-*O*-glucoside shows antioxidant activity [43]; and lariciresinol-4-*O*-β-D-glucopyranoside shows anti-inflammatory and antiviral effects [44]. It can also be assumed that not only the main chemical constituents with high content but also all of these compounds identified in the Lanqin oral liquid have the probability of being bioactive constituents. Moreover, this study provides the necessary basis for further research on the functional spectrum of Lanqin oral liquid, which may explore and elucidate the therapeutic basis and the possible action mechanism of Lanqin oral liquid on the functions of clearing heat and detoxification, liyan, and detumescence effects.

## 3. Materials and Methods

### 3.1. Chemicals and Reagents

Lanqin oral liquid (lot no. 19122821) was purchased from Yangtze River Pharmaceutical Group (Jiangsu, China). Reference compounds (purity > 98%) of chlorogenic acid, caffeic acid, scutellarin, baicalin, palmatine, wogonoside, apigenin, baicalein, wogonin, indirubin, and indigo were purchased from Chengdu Must Bio-Technology Co., Ltd. (Chengdu, China). Acetonitrile and formic acid of HPLC grade were obtained from Fisher Scientific (Fair Lawn, NJ, USA). Purified water was purchased from Hangzhou Wahaha Corporation (Hangzhou, China). All other chemical reagents used in this study were of analytical grade.

### 3.2. Sample Preparation

1 mL of Lanqin oral liquid was accurately measured and diluted with 1 mL of water. The mixture was vortexed for 3 min, and then the solution was prepared by diluting 5-fold with ultrapure water. The mixture was vortexed for 3 min, then centrifuged at 12,000 rpm for 10 min. The supernatant was collected and filtered through a 0.22 µm filter membrane before UHPLC-FT-ICR-MS/analysis.

### 3.3. Liquid Chromatography

An Agilent 1260 UHPLC system (Agilent, Santa Clara, CA, USA) was used for the analysis. Chromatographic separation was performed on an ACQUITY UPLC HSS T3 column (2.1 mm × 100 mm, 1.8 µm, Waters, Wexford, Ireland) with an ACQUITY UPLC HSS T3 VanGuard Pre-Column (2.1 mm × 5 mm, 1.8 µm, Waters, Wexford, Ireland) kept at 35 °C. With a flow rate of 0.30 mL/min, the mobile phase was a gradient system consisting of water infused with 0.1% formic acid (A) and acetonitrile (B), with the gradient as follows: 0–2 min, 8–20% B; 2–20 min, 20–50% B; 20–26 min, 50–98% B; 26–33 min, an isocratic elution of B. The injection volume was set at 10 µL.

### 3.4. Mass Spectrometry

Mass spectrometric detection was carried out on a Solarix 7.0T FT-ICR mass spectrometer (Bruker, Bremen, Germany) coupled with an electrospray ionization (ESI) interface. The full-scan MS data acquired in both positive and negative modes scanned from 100 to 1200 Da. The capillary voltage was 4500 V, the endplate offset was 500 V, dry gas flow was 8 L/min, dry gas temperature was 200 °C, and nebulizer gas pressure was 4 bar. The collision gas and nebulizing gas were high-purity argon (Ar) and high-purity nitrogen (N_2_), respectively. Full-scan MS data were acquired over an *m*/*z* range of 100–1200 Da. And the collision energy were set from 10 to 40 eV for fragmentation information. Bruker Compass Hystar (version 4.1, Bruker Daltonics, Bremen, Germany) and Fourier Transform Mass Spectrometer Control (version 2.1, Bruker Daltonics, Bremen, Germany) were used for instrument control and data acquisition. Data Analysis Software (version 4.4, Bruker Daltonics, Bremen, Germany) was used for data analysis.

## 4. Conclusions

In our study, a rapid, sensitive, and specific analytical method using UHPLC-FT-ICR-MS/MS was successfully applied to separate and identify chemical constituents of Lanqin oral liquid. A total of 441 compounds were identified or tentatively characterized in Lanqin oral liquid, revealing that flavonoids, alkaloids, terpenoids, organic acids, and phenylpropanoids were the main components. The applied UHPLC-FT-ICR-MS method with the advantages of good separation, high sensitivity, high resolution, and high accuracy for the detection of chemical constituents in Lanqin oral liquid provided comprehensive chemical information on Lanqin oral liquid and could aid in quality control and an understanding of the pharmacodynamic material basis and thus promote the development of Lanqin oral liquid.

## Figures and Tables

**Table 1:** Flavonoids identified in Lanqin oral liquid by UHPLC-FT-ICR MS.

No.	RT (min)	Identification	[M-H]^+^				[M-H]^−^				Product Ion (*m*/*z*)
			Observed	Calculated	Error (ppm)	Formula	Observed	Calculated	Error (ppm)	Formula	
F1	6.02	Apigenin-6,8-di-*C*-glucoside	-	-	-	-	593.15122	593.15119	−0.04	C_27_H_29_O_15_	(−): 503, 473, 383, 353
F2	6.96	Schaftoside	565.15541	565.15518	−0.41	C_26_H_29_O_14_	563.14072	563.14063	−0.16	C_26_H_27_O_14_	(−): 545, 515, 503, 485, 473, 455, 443, 437, 425, 413, 407, 395, 383, 365, 353, 337, 335, 325, 323, 311, 297
F3	7.16	Isoschaftoside	565.15754	565.15518	−4.17	C_26_H_29_O_14_	563.14032	563.14063	0.55	C_26_H_27_O_14_	(−): 545, 515, 503, 485, 473, 455, 443, 437, 425, 413, 407, 395, 383, 365, 353, 337, 335, 325, 323, 311, 297
F4	7.19	Viscidulin III-2′-*O*-glucoside isomer	509.13073	509.12897	−3.46	C_23_H_25_O_13_	507.11404	507.11441	0.73	C_23_H_23_O_13_	(+): 347
F5	7.34	Chrysin-6,8-di-*C*-glucoside	579.17156	579.17083	−1.25	C_27_H_31_O_14_	577.15585	577.15628	0.75	C_27_H_29_O_14_	(−): 487, 457, 409, 397, 379, 367, 349, 337, 321, 319, 309, 295, 281, 267
F6	7.82	Rutin	611.16025	611.16066	0.67	C_27_H_31_O_16_	609.14542	609.14611	1.13	C_27_H_29_O_16_	(+): 465, 303
F7	7.90	Chrysin 6-*C*-glucoside 8-*C*-arabinoside	549.15924	549.16027	1.87	C_26_H_29_O_13_	547.14467	547.14571	1.92	C_26_H_27_O_13_	(+): 531, 513, 495, 483, 477, 465, 459, 447, 441, 429, 423, 417, 411, 405, 399, 393, 387, 375, 363, 357, 351, 345, 339, 333, 321, 309, 291, 279
F8	7.95	Isovitexin	433.11379	433.11292	−1.99	C_21_H_21_O_10_	-	-	-	-	(+): 415, 397, 379, 367, 361, 351, 349, 337, 323, 313, 309, 295, 283
F9	8.09	Tetrahydroxyflavanone	289.07117	289.07066	−1.77	C_15_H_13_O_6_	287.05575	287.05611	1.26	C_15_H_11_O_6_	(+): 153
F10	8.10	Isoquercitrin	465.10341	465.10275	−1.41	C_21_H_21_O_12_	463.08779	463.08820	0.88	C_21_H_19_O_12_	(+): 303
F11	8.15	Kaempferol-3-*O*-rhamnose-7-*O*-glucose	-	-	-	-	593.15122	593.15119	−0.04	C_27_H_29_O_15_	(−): 285
F12	8.22	Scutellarin *	463.08755	463.08710	−0.96	C_21_H_19_O_12_	461.07229	461.07255	0.56	C_21_H_17_O_12_	(−): 285
F13	8.24	Hyperin	465.10326	465.10275	−1.10	C_21_H_21_O_12_	463.08787	463.08820	0.71	C_21_H_19_O_12_	(+): 303
F14	8.26	2′,3′,4′,5,7-pentahydroxyflavone	303.04997	303.04993	−0.12	C_15_H_11_O_7_	301.03517	301.03538	0.70	C_15_H_9_O_7_	(+): 153
F15	8.34	Viscidulin III-2′-*O*-glucoside	509.12956	509.12897	−1.17	C_23_H_25_O_13_	507.11389	507.11441	1.04	C_23_H_23_O_13_	(+): 347
F16	8.37	Luteolin-7-*O*-rutinoside	-	-	-	-	593.14991	593.15119	2.17	C_27_H_29_O_15_	(−): 285
F17	8.43	Chrysin-6-*C*-arabinoside-8-*C*-glucoside	549.15943	549.16027	1.52	C_26_H_29_O_13_	547.14480	547.14571	1.68	C_26_H_27_O_13_	(−): 487,457, 427, 409, 397, 391, 379, 373, 367, 363, 349, 337, 333, 331, 321, 319, 309, 307, 295, 293, 281
F18	8.56	Tetrahydroxyflavanone	289.07094	289.07066	−0.95	C_15_H_13_O_6_	287.05535	287.05611	2.65	C_15_H_11_O_6_	(+): 153
F19	8.57	Quercetin 7-*O*-glucoside	465.10325	465.10275	−1.07	C_21_H_21_O_12_	463.08776	463.08820	0.94	C_21_H_19_O_12_	(−): 301, 151
F20	8.62	Chrysin-6-*C*-arabinoside-8-*C*-glucoside isomer	549.16023	549.16027	0.07	C_26_H_29_O_13_	547.14503	547.14571	1.25	C_26_H_27_O_13_	(−): 487,457, 427, 409, 397, 391, 379, 373, 367, 363, 349, 337, 333, 331, 321, 319, 309, 307, 295, 293, 281
F21	8.71	Nicotiflorin	595.16729	595.16575	−2.60	C_27_H_31_O_15_	593.15093	593.15119	0.45	C_27_H_29_O_15_	(+): 287
F22	8.92	Kaempferol-3-*O*-rhamnose-7-*O*-glucose isomer	595.16675	595.16575	−1.69	C_27_H_31_O_15_	593.15039	593.15119	1.35	C_27_H_29_O_15_	(−): 285
F23	9.04	5,6′-dihydroxy-6,7-dimethoxyflavone-2′-*O*-glucoside	493.13457	493.13405	−1.06	C_23_H_25_O_12_	491.11905	491.11950	0.91	C_23_H_23_O_12_	(−): 329
F24	9.16	Quercitrin	449.10890	449.10784	−2.37	C_21_H_21_O_11_	447.09288	447.09329	0.91	C_21_H_19_O_11_	(−): 301
F25	9.16	Oroxin B	595.16702	595.16575	−2.14	C_27_H_31_O_15_	593.15081	593.15119	0.65	C_27_H_29_O_15_	(−): 269
F26	9.40	Chrysin 8-*C*-glucoside	417.11859	417.11801	−1.40	C_21_H_21_O_9_	415.10314	415.10346	0.77	C_21_H_19_O_9_	(+): 399, 381, 363, 351, 339, 335, 327, 321, 307, 297, 279, 267
F27	9.43	Apigenin-7-*O*-glucoside	433.11325	433.11292	−0.75	C_21_H_21_O_10_	431.09772	431.09837	1.51	C_21_H_19_H_10_	(+): 271
F28	9.49	Linarin	-	-	-	-	591.17133	591.17193	1.02	C_28_H_31_O_14_	(−): 283
F29	9.55	Isorhamnetin-3-*O*-glucoside	479.11935	479.11840	−1.98	C_22_H_23_O_12_	477.10352	477.10385	0.70	C_22_H_21_O_12_	(+): 317
F30	9.58	Hesperidin	-	-	-	-	609.18261	609.18249	−0.19	C_28_H_33_O_15_	(−): 301
F31	9.72	5,2′,6′-dihydroxy-6,7,8-trimethoxyflavone-2′-*O*-glucoside	523.14492	523.14462	−0.59	C_24_H_27_O_13_	521.12952	521.13006	1.04	C_24_H_25_O_13_	(−): 359, 344, 329
F32	9.84	Kaempferide 3-glucuronide	477.10324	477.10275	−1.02	C_22_H_21_O_12_	475.08784	475.08820	0.76	C_22_H_19_O_12_	(−): 299, 284
F33	9.86	Astragalin	449.10851	449.10784	−1.50	C_21_H_21_O_11_	447.09287	447.09329	0.93	C_21_H_19_O_11_	(+): 287
F34	9.95	Viscidulin I	303.05004	303.04993	−0.37	C_15_H_11_O_7_	301.03513	301.03538	0.81	C_15_H_9_O_7_	(−): 151
F35	10.01	Chrysoeriol-5-*O*-β-D-glucuronide	477.10317	477.10275	−0.88	C_22_H_21_O_12_	475.08768	475.08820	1.09	C_22_H_19_O_12_	(−): 299
F36	10.11	Scutellarein-4′-methyl ether-*O*-glucuronide	477.10272	477.10275	0.06	C_22_H_21_O_12_	475.08773	475.08820	0.99	C_22_H_19_O_12_	(−): 443, 284, 173, 155
F37	10.27	Scutellarein	287.05510	287.05501	−0.29	C_15_H_11_O_6_	285.04023	285.04046	0.81	C_15_H_9_O_6_	(+): 269, 241
F38	10.27	2′,6′,7-trihydroxy-5-methoxyflavanone	303.08649	303.08631	−0.58	C_16_H_15_O_6_	301.07155	301.07176	0.72	C_16_H_13_O_6_	(+): 167, 152, 123
F39	10.60	Syringetin	347.07655	347.07614	−1.17	C_17_H_15_O_8_	345.06107	345.06159	1.50	C_17_H_13_O_8_	(+): 332
F40	10.93	Dihydrokaempferol	289.07086	289.07066	−0.67	C_15_H_13_O_6_	287.05588	287.05611	0.82	C_15_H_11_O_6_	(+): 271, 153
F41	10.94	Trihyroxy-dimethoxy flavone-*O*-glucuronide	507.11405	507.11332	−1.44	C_23_H_23_O_13_	505.09814	505.09876	1.23	C_23_H_21_O_13_	(+): 331
F42	11.05	Baicalin *	447.09220	447.09219	−0.03	C_21_H_19_O_11_	445.07677	445.07763	1.94	C_21_H_17_O_11_	(−): 269
F43	11.06	Fisetin	287.05504	287.05501	−0.09	C_15_H_11_O_6_	-	-	-	-	(+): 269, 258, 241, 231, 223, 213
F44	11.12	Viscidulin III	347.07619	347.07614	−0.14	C_17_H_15_O_8_	345.06120	345.06159	1.14	C_17_H_13_O_8_	(+): 332, 314, 286
F45	11.20	Oroxin A	433.11331	433.11292	−0.89	C_21_H_21_O_10_	431.09794	431.09837	1.01	C_21_H_19_O_10_	(+): 271
F46	11.24	Chrysin 6-*C*-glucoside	417.11836	417.11801	−0.85	C_21_H_21_O_9_	415.10304	415.10346	1.01	C_21_H_19_O_9_	(+): 381, 363, 351, 345, 335, 327, 333, 321, 307, 297, 293, 279, 267
F47	11.33	Tetrahydroxyflavanone	289.07083	289.07066	−0.56	C_15_H_13_O_6_	287.05592	287.05611	0.68	C_15_H_11_O_6_	(+): 153
F48	11.38	Trihyroxy-dimethoxy flavone-*O*-glucuronide	507.11429	507.11332	−1.91	C_23_H_23_O_13_	505.09829	505.09876	0.94	C_23_H_21_O_13_	(+): 331
F49	11.56	Tetrahydroxyflavanone	289.07097	289.07066	−1.05	C_15_H_13_O_6_	287.05586	287.05611	0.89	C_15_H_11_O_6_	(+): 153
F50	11.67	Baicalin ethyl ester or isomer	475.12476	475.12349	−2.68	C_23_H_23_O_11_	473.10838	473.10894	1.17	C_23_H_21_O_11_	(+): 271
F51	11.78	Chrysoeriol-7-*O*-β-D-glucuronide	477.10404	477.10275	−2.71	C_22_H_21_O_12_	475.08786	475.08820	0.72	C_22_H_19_O_12_	(−): 299, 284
F52	11.88	5,6-dihydroxyl flavanone-7-*O*-glucuronide	449.10885	449.10784	−2.25	C_21_H_21_O_11_	447.09264	447.09329	1.44	C_21_H_19_O_11_	(−): 271
F53	11.89	Trihydroxyflavanone	273.07602	273.07575	−0.97	C_15_H_13_O_5_	271.06090	271.06120	1.08	C_15_H_11_O_5_	(+): 153
F54	12.08	Oroxin A isomer	-	-	-	-	431.09797	431.09837	0.92	C_21_H_19_O_10_	(−): 269
F55	12.08	Oroxylin A 7-*O*-β-D-glucoside	447.13030	447.12857	−3.85	C_22_H_23_O_10_	445.11344	445.11402	1.31	C_22_H_21_O_10_	(−): 268
F56	12.16	Dihydroxyl flavanone-*O*-glucuronide	449.10921	449.10784	−3.06	C_21_H_21_O_11_	447.09263	447.09329	1.46	C_21_H_19_O_11_	(−): 271
F57	12.26	Norwogonin-7-*O*-glucuronide	447.09270	447.09219	−1.14	C_21_H_19_O_11_	445.07679	445.07763	1.90	C_21_H_17_O_11_	(−): 269
F58	12.38	2′-hydroxyformononetin	285.07651	285.07575	−2.66	C_16_H_13_O_5_	283.06083	283.06120	1.29	C_16_H_11_O_5_	(+): 270
F59	12.42	Fisetin isomer	287.05580	287.05501	−2.74	C_15_H_11_O_6_	285.04020	285.04046	0.91	C_15_H_9_O_6_	(+): 241
F60	12.50	Hydroxyl oroxylin-A-7-*O*-glucuronide	477.10448	477.10275	−3.62	C_22_H_21_O_12_	475.08756	475.08820	1.35	C_22_H_19_O_12_	(−): 299, 284
F61	12.72	Norwogonin-8-*O*-glucuronide isomer	447.09344	447.09219	−2.81	C_21_H_19_O_11_	445.07693	445.07763	1.57	C_21_H_17_O_11_	(−): 269
F62	12.74	Dihydroxyflavone-*O*-glucopyranoside	417.11905	417.11801	−2.50	C_21_H_21_O_9_	415.10311	415.10346	0.83	C_21_H_19_O_9_	(+): 255
F63	12.75	Kaempferin	433.11420	433.11292	−2.94	C_21_H_21_O_10_	431.09791	431.09837	1.06	C_21_H_19_O_10_	(+): 271
F64	12.79	Tricin 7-*O*-glucoside	-	-	-	-	491.11895	491.11950	1.11	C_23_H_23_O_12_	(−): 329, 313
F65	12.82	Quercetin	-	-	-	-	301.03524	301.03538	0.45	C_15_H_9_O_7_	(−): 151
F66	12.97	Oroxylin A-7-*O*-glucuronide	461.10755	461.10784	0.62	C_22_H_21_O_11_	459.09257	459.09329	1.55	C_22_H_19_O_11_	(+): 285, 270
F67	13.00	Chrysin-7-*O*-glucuronide	431.09825	431.09727	−2.26	C_21_H_19_O_10_	429.08215	429.08272	1.34	C_21_H_17_O_10_	(−): 253
F68	13.08	Hydroxyl wogonoside	477.10320	477.10275	−0.94	C_22_H_21_O_12_	475.08742	475.08820	1.65	C_22_H_19_O_12_	(−): 299
F69	13.35	Norwogonin-8-*O*-glucuronide	447.09296	447.09219	−1.73	C_21_H_19_O_11_	445.07720	445.07763	0.97	C_21_H_17_O_11_	(−): 269
F70	13.48	Oroxin A isomer	433.11342	433.11292	−1.15	C_21_H_21_O_10_	431.09809	431.09837	0.64	C_21_H_19_H_10_	(+): 271
F71	13.62	Wogonoside *	461.10765	461.10784	0.40	C_22_H_21_O_11_	459.09255	459.09329	1.60	C_22_H_19_O_11_	(−): 283, 268
F72	13.84	Dihydrooroxylin A	287.09159	287.09140	−0.65	C_16_H_15_O_5_	285.07658	285.07685	0.94	C_16_H_13_O_5_	-
F73	13.85	(2S)-5-hydroxy-6-methoxyflavanone 7-*O*-*β*-D-glucuronide	463.12401	463.12349	−1.12	C_22_H_23_O_11_	461.10839	461.10894	1.18	C_22_H_21_O_11_	(−): 285, 270
F74	13.91	Trihydroxy-methoxyflavanone	303.08658	303.08631	−0.89	C_16_H_15_O_6_	301.07153	301.07176	0.78	C_16_H_13_O_6_	(+): 147, 135
F75	13.94	Oroxin A isomer	433.11357	433.11292	−1.49	C_21_H_21_O_10_	431.09808	431.09837	0.66	C_21_H_19_H_10_	(+): 271
F76	13.97	5,7-dihydroxy-6,8-dimethoxyflavone-7-*O*-glucuronide	491.11836	491.11840	0.09	C_23_H_23_O_12_	489.10335	489.10385	1.03	C_23_H_21_O_12_	(+): 315, 300, 285
F77	14.08	Viscidulin II isomer	331.08144	331.08123	−0.63	C_17_H_15_O_7_	329.06652	329.06668	0.48	C_17_H_13_O_7_	(+): 316, 301
F78	14.08	(2S)-5-hydroxy-6-methoxyflavanone 7-*O*-β-D-glucuronide isomer	463.12409	463.12349	−1.30	C_22_H_23_O_11_	461.10851	461.10894	0.93	C_22_H_21_O_11_	(−): 285, 270
F79	14.10	Baicalin ethyl ester or isomer	475.12392	475.12349	−0.92	C_23_H_23_O_11_	473.10881	473.10894	0.26	C_23_H_21_O_11_	(+): 271
F80	14.20	Skullcapflavone II isomer	375.10761	375.10744	−0.45	C_19_H_19_O_8_	373.09286	373.09289	0.08	C_19_H_17_O_8_	(+): 360, 345, 327
F81	14.50	Mosloflavone isomer	299.09137	299.09140	0.10	C_17_H_15_O_5_	297.07684	297.07685	0.02	C_17_H_13_O_5_	(+): 238
F82	14.58	Epimedoside C or isomer	517.17064	517.17044	−0.39	C_26_H_29_O_11_	515.15542	515.15589	0.90	C_26_H_27_O_11_	(+): 355
F83	14.68	Chrysosplenin or isomer	537.16057	537.16027	−0.57	C_25_H_29_O_13_	-	-	-	-	(+): 375, 345
F84	14.80	Baicalin ethyl ester or isomer	475.12353	475.12349	−0.09	C_23_H_23_O_11_	473.10828	473.10894	1.38	C_23_H_21_O_11_	(+): 271
F85	14.82	Phellamurin	519.18583	519.18609	0.50	C_26_H_31_O_11_	563.17651	563.17701	0.89 ^a^	C_27_H_31_O_13_	(−): 517
F86	14.88	Trihydroxy-methoxyflavanone	303.08625	303.08631	0.21	C_16_H_15_O_6_	301.07157	301.07176	0.64	C_16_H_13_O_6_	(+): 147, 135
F87	14.95	Alpinetin	271.09657	271.09649	−0.32	C_16_H_15_O_4_	-	-	-	-	(+): 167
F88	15.06	Tenaxin II	301.07046	301.07066	0.69	C_16_H_13_O_6_	299.05588	299.05611	0.77	C_16_H_11_O_6_	(−): 284
F89	15.14	Apigenin *	271.05992	271.06010	0.65	C_15_H_11_O_5_	269.04529	269.04555	0.96	C_15_H_9_O_5_	(−): 237, 225, 201, 175, 151, 149
F90	15.14	5,2′,5′-trihydroxy-6,7,8-trimethoxyflavone or isomer	361.09173	361.09179	0.17	C_18_H_17_O_8_	359.07692	359.07724	0.90	C_18_H_15_O_8_	(+): 346, 331, 328, 313
F91	15.15	Kaempferol isomer	287.05503	287.05501	−0.06	C_15_H_11_O_6_	285.04027	285.04046	0.67	C_15_H_9_O_6_	(−): 227, 211
F92	15.23	5,2′,6′-dihydroxy-6,7,8-trimethoxyflavone-2′-*O*-glucoside isomer	523.14464	523.14462	−0.05	C_24_H_27_O_13_	521.12962	521.13006	0.86	C_24_H_25_O_13_	(+): 361
F93	15.31	Viscidulin II isomer	331.08119	331.08123	0.12	C_17_H_15_O_7_	329.06642	329.06668	0.78	C_17_H_13_O_7_	(+): 316, 301
F94	15.47	Tectorigenin	301.07062	301.07066	0.14	C_16_H_13_O_6_	299.05586	299.05611	0.86	C_16_H_11_O_6_	(+): 286
F95	15.52	Kaempferol	287.05507	287.05501	−0.18	C_15_H_11_O_6_	285.04023	285.04046	0.80	C_15_H_9_O_6_	(+): 269, 258, 241, 231, 213
F96	15.73	Norwogonin	271.06005	271.06010	0.18	C_15_H_11_O_5_	269.04531	269.04555	0.89	C_15_H_9_O_5_	(+): 253, 225
F97	15.78	Viscidulin II isomer	331.08125	331.08123	−0.07	C_17_H_15_O_7_	329.06661	329.06668	0.21	C_17_H_13_O_7_	(+): 316
F98	15.85	Baicalin ethyl ester or isomer	475.12347	475.12349	0.05	C_23_H_23_O_11_	473.10934	473.10894	−0.86	C_23_H_21_O_11_	(+): 271
F99	16.00	Trihydroxyflavanone	273.07572	273.07575	0.12	C_15_H_13_O_5_	271.06100	271.06120	0.74	C_15_H_11_O_5_	(+): 153
F100	16.07	4′-hydroxyl wogonin	301.07044	301.07066	0.75	C_16_H_13_O_6_	299.05577	299.05611	1.15	C_16_H_11_O_6_	(+): 286
F101	16.10	5,2′,5′-trihydroxy-6,7,8-trimethoxyflavone or isomer	361.09207	361.09179	−0.77	C_18_H_17_O_8_	359.07696	359.07724	0.78	C_18_H_15_O_8_	(+): 346, 331, 328, 313
F102	16.16	Viscidulin II	331.08127	331.08123	−0.13	C_17_H_15_O_7_	329.06645	329.06668	0.69	C_17_H_13_O_7_	(+): 316
F103	16.32	Baicalein *	271.05990	271.06010	0.72	C_15_H_11_O_5_	269.04525	269.04555	1.11	C_15_H_9_O_5_	(+): 253, 225
F104	16.35	Fisetin isomer	287.05543	287.05501	−1.45	C_15_H_11_O_6_	285.04034	285.04046	0.42	C_15_H_9_O_6_	(+): 241
F105	16.36	Skullcapflavone II isomer	375.10807	375.10744	−1.66	C_19_H_19_O_8_	373.09286	373.09289	0.08	C_19_H_17_O_8_	(+): 360, 345, 327
F106	16.40	Kaempferide isomer	301.07060	301.07066	0.21	C_16_H_13_O_6_	299.05586	299.05611	0.86	C_16_H_11_O_6_	(+): 286
F107	16.40	5,2′,5′-trihydroxy-6,7,8-trimethoxyflavone or isomer	361.09177	361.09179	0.07	C_18_H_17_O_8_	359.07724	359.07724	0.00	C_18_H_15_O_8_	(+): 346, 331, 328, 313
F108	16.68	Trihydroxyflavanone	273.07580	273.07575	−0.19	C_15_H_13_O_5_	271.06095	271.06120	0.90	C_15_H_11_O_5_	(+): 153
F109	16.75	Tricin	331.08117	331.08123	0.19	C_17_H_15_O_7_	329.06638	329.06668	0.92	C_17_H_13_O_7_	(−): 314, 299
F110	17.14	5,8,2′-trihydroxy-7-methoxyflavone	301.07071	301.07066	−0.14	C_16_H_13_O_6_	299.05608	299.05611	0.12	C_16_H_11_O_6_	(+): 286
F111	17.23	5,8,2′-trihydroxy-6,7-dimethoxyflavone	331.08112	331.08123	0.32	C_17_H_15_O_7_	329.06661	329.06668	0.21	C_17_H_13_O_7_	(+): 316, 301
F112	17.40	Trihydroxy-methoxyflavanone	303.08621	303.08631	0.36	C_16_H_15_O_6_	301.07156	301.07176	0.68	C_16_H_13_O_6_	(+): 147, 135
F113	18.46	5,7-dihydroxy-8,2′,3′,6-tetramethoxyflavone or isomer	391.10200	391.10236	0.92	C_19_H_19_O_9_	389.08755	389.08781	0.66	C_19_H_17_O_9_	(+): 361
F114	18.63	Trimethoxyflavone	313.10681	313.10705	0.76	C_18_H_17_O_5_	-	-	-	-	(+): 298, 283
F115	18.70	Trihydroxy-dimethoxyflavone	331.08108	331.08123	0.46	C_17_H_15_O_7_	329.06646	329.06668	0.66	C_17_H_13_O_7_	(+): 316, 301
F116	19.68	Dihydrooroxylin A isomer	287.09142	287.09140	−0.07	C_16_H_15_O_5_	285.07672	285.07685	0.46	C_16_H_13_O_5_	-
F117	19.71	5,7,3′-trihydroxy-6,4′,5′-trimethoxyflavone	361.09172	361.09179	0.21	C_18_H_17_O_8_	359.07710	359.07724	0.39	C_18_H_15_O_8_	(−): 344, 329, 314
F118	20.12	Rivularin	345.09650	345.09688	1.10	C_18_H_17_O_7_	343.08212	343.08233	0.59	C_18_H_15_O_7_	(−): 328, 313, 298, 285, 270
F119	20.41	Wogonin *	285.07536	285.07575	1.35	C_16_H_13_O_5_	283.06071	283.06120	1.72	C_16_H_11_O_5_	(+): 270
F120	20.61	Amentoflavone isomer	539.09726	539.09727	0.02	C_30_H_19_O_10_	537.08211	537.08272	1.14	C_30_H_17_O_10_	(−): 391, 373, 268, 245
F121	20.63	Chrysin	255.06516	255.06519	0.11	C_15_H_11_O_4_	253.05045	253.05063	0.73	C_15_H_9_O_4_	(−): 209
F122	20.78	Skullcapflavone II isomer	375.10723	375.10744	0.58	C_19_H_19_O_8_	373.09290	373.09289	−0.03	C_19_H_17_O_8_	(+): 360, 345, 327
F123	20.82	Dihydrooroxylin A isomer	287.09149	287.09140	−0.30	C_16_H_15_O_5_	285.07676	285.07685	0.30	C_16_H_13_O_5_	-
F124	20.88	Skullcapflavone I	315.08618	315.08631	0.44	C_17_H_15_O_6_	313.07148	313.07176	0.89	C_17_H_13_O_6_	(−): 298, 283, 255, 239, 227, 211, 201, 199, 183, 173, 155
F125	21.21	Skullcapflavone II	375.10743	375.10744	0.03	C_19_H_19_O_8_	373.09252	373.09289	0.99	C_19_H_17_O_8_	(−): 358, 343, 328, 315, 300, 285, 272, 269, 257, 213
F126	21.33	Oroxylin A	285.07557	285.07575	0.62	C_16_H_13_O_5_	283.06108	283.06120	0.43	C_16_H_11_O_5_	(+): 270
F127	21.53	Dihydrooroxylin A isomer	287.09137	287.09140	0.10	C_16_H_15_O_5_	285.07677	285.07685	0.25	C_16_H_13_O_5_	-
F128	21.59	5,8-dihydroxy-6,7-dimethoxyflavone	315.08612	315.08631	0.63	C_17_H_15_O_6_	313.07150	313.07176	0.84	C_17_H_13_O_6_	(−): 298, 283, 255, 239, 227, 211, 183
F129	21.67	Amentoflavone isomer	539.09716	539.09727	0.20	C_30_H_19_O_10_	537.08265	537.08272	0.13	C_30_H_17_O_10_	-
F130	22.60	5,2′-dihydroxy-6,7,8-trimethoxyflavone	345.09651	345.09688	1.08	C_18_H_17_O_7_	343.08199	343.08233	0.98	C_18_H_15_O_7_	(−): 328, 313, 298, 295, 270
F131	23.71	7-*O*-methylscutellarein-*O*-baicalein	553.11268	553.11292	0.44	C_31_H_21_O_10_	551.09789	551.09837	0.87	C_31_H_19_O_10_	(−): 281, 269
F132	24.09	Mosloflavone isomer	299.09128	299.09140	0.40	C_17_H_15_O_5_	-	-	-	-	(+): 284
F133	24.77	Moslosooflavone	299.09130	299.09140	0.32	C_17_H_15_O_5_	-	-	-	-	(+): 284, 266, 238
F134	25.46	Mosloflavone	299.09133	299.09140	0.22	C_17_H_15_O_5_	-	-	-	-	(+): 284, 255

^a^: [M + HCOO]^−^. -: not detected. * Compared with a reference standard.

**Table 2:** Alkaloids identified in Lanqin oral liquid by UHPLC-FT-ICR MS.

No.	RT (min)	Identification	[M-H]^+^				[M-H]^−^				Product Ion (*m*/*z*)
			Observed	Calculated	Error (ppm)	Formula	Observed	Calculated	Error (ppm)	Formula	
A1	1.62	Candicine	180.13838	180.13829	−0.51	C_11_H_18_NO^+^	-	-	-	-	(+): 121
A2	2.51	*N*-methylhigenamine 7-glucopyranoside isomer	448.19671	448.19659	−0.25	C_23_H_30_NO_8_	-	-	-	-	(+): 286
A3	3.43	Juziphine isomer	300.15938	300.15942	0.13	C_18_H_22_NO_3_	-	-	-	-	(+): 107
A4	3.99	*N*-methylhigenamine 7-glucopyranoside isomer	448.19649	448.19659	0.23	C_23_H_30_NO_8_	-	-	-	-	(+): 286
A5	4.05	Laudanosoline 6-mether-4′-*O*-glucopyranoside isomer	478.20728	478.20716	−0.26	C_24_H_32_NO_9_	-	-	-	-	(+): 316
A6	4.37	Higenamine	272.12795	272.12812	0.64	C_16_H_18_NO_3_	-	-	-	-	(+): 107
A7	4.42	Laudanosoline 6-mether-4′-*O*-glucopyranoside	478.20735	478.20716	−0.40	C_24_H_32_NO_9_	-	-	-	-	(+): 316
A8	4.43	Juziphine-4′-*O*-glucopyranoside	462.21227	462.21224	−0.06	C_24_H_32_NO_8_	-	-	-	-	(+): 300
A9	4.57	Govadine	328.15405	328.15433	0.87	C_19_H_22_NO_4_	-	-	-	-	(+): 178
A10	4.71	Coclaurine isomer	286.14372	286.14377	0.16	C_17_H_20_NO_3_	-	-	-	-	-
A11	5.07	Isaindigodione isomer	327.13367	327.13393	0.80	C_18_H_19_N_2_O_4_	-	-	-	-	(+): 201
A12	5.16	Protosinomenine-3′-*O*-glucopyranoside	492.22311	492.22281	−0.62	C_25_H_34_NO_9_	-	-	-	-	(+): 330, 299, 192
A13	5.28	Unknown	328.15414	328.15433	0.58	C_19_H_22_NO_4_	-	-	-	-	(+): 192
A14	5.34	Corypalmine	342.17025	342.16998	−0.78	C_20_H_24_NO_4_	-	-	-	-	(+): 297
A15	5.37	Isaindigodione	327.13345	327.13393	1.47	C_18_H_19_N_2_O_4_	-	-	-	-	(+): 201
A16	5.41	Lotusine	314.17516	314.17507	−0.29	C_19_H_24_NO_3_	-	-	-	-	(+): 269, 237
A17	5.43	lndole-3-acetonitrile-6-*O*-β-D-glucopyranoside	335.12407	335.12376	−0.92	C_16_H_19_N_2_O_6_	379.11463	379.11469	0.16 ^a^	C_17_H_19_N_2_O_8_	(−): 333, 306, 144
A18	5.46	Protosinomenine isomer	330.17006	330.16998	−0.23	C_19_H_24_NO_4_	-	-	-	-	-
A19	5.51	(E)-3-(3′,5′-dimethoxy-4′-hydroxy-benzylidene)-2-indolinone	298.10770	298.10738	−1.06	C_17_H_16_NO_4_	-	-	-	-	-
A20	5.57	Protosinomenine-3′-*O*-glucopyranoside isomer	492.22283	492.22281	−0.04	C_25_H_34_NO_9_	-	-	-	-	(+): 300
A21	5.63	Indican	296.11296	296.11286	−0.33	C_14_H_18_NO_6_	340.10358	340.10379	0.61 ^a^	C_15_H_18_NO_8_	(−): 294, 161, 132
A22	5.65	Unknown	356.14910	356.14925	0.43	C_20_H_22_NO_5_	-	-	-	-	(+): 206
A23	5.74	3,4-dihydro-[(4-hydroxyphenyl)methyl]-7-methoxy-2-methyl-8-isoquinolinol	298.14366	298.14377	0.35	C_18_H_20_NO_3_	-	-	-	-	(+): 283, 282, 254
A24	5.80	Magnocurarine	314.17486	314.17507	0.66	C_19_H_24_NO_3_	-	-	-	-	(+): 269, 237
A25	5.81	2,3-dihydro-1H-pyrrolo[2,1-c][1,4]benzodiazepine-5,11(10H,11ah)-dione	217.09734	217.09715	−0.85	C_12_H_13_N_2_O_2_	-	-	-	-	(+): 144
A26	5.85	Juziphine-4′-*O*-glucopyranoside	462.21275	462.21224	−1.10	C_24_H_32_NO_8_	-	-	-	-	(+): 300
A27	5.95	Unknown	356.14938	356.14925	−0.36	C_20_H_22_NO_5_	-	-	-	-	(+): 206
A28	6.05	Phellodendrine	342.16909	342.16998	2.62	C_20_H_24_NO_4_	-	-	-	-	(+): 192
A29	6.15	*N*-methylhigenamine 7-glucopyranoside	448.19537	448.19659	2.72	C_23_H_30_NO_8_	446.18187	446.18204	0.37	C_23_H_28_NO_8_	(+): 286, 255
A30	6.20	Anthranilic acid-7-*O*-β-D-glucopyranose ester	300.10827	300.10778	−1.64	C_13_H_18_NO_7_	-	-	-	-	(+): 282, 264, 246, 138
A31	6.40	Tembetarine	344.18565	344.18563	−0.03	C_20_H_26_NO_4_	-	-	-	-	(+): 175, 137
A32	6.43	8-*O*-methyloblongine	328.19083	328.19072	−0.33	C_20_H_26_NO_3_	-	-	-	-	(+): 313, 283
A33	6.46	(1S)-1,2,3,4-tetrahydro-7-hydroxy-1-[(4-hydroxybenzyl)methyl]-2,2-dimethyl-8-O-isoquinolinyl-[3-hydroxy-3-methylglutaryl]-β-D-glucopyranoside	592.23893	592.23885	−0.13	C_29_H_38_NO_12_	590.22428	590.22430	0.03	C_29_H_36_NO_12_	(+): 530, 490, 448, 286
A34	6.52	Magnoflorine	342.16988	342.16998	0.31	C_20_H_24_NO_4_	-	-	-	-	(+): 297, 282, 265, 237
A35	6.61	Piperlonguminine	274.14417	274.14377	−1.46	C_16_H_20_NO_3_	-	-	-	-	(+): 256, 227
A36	6.66	Unknown	312.12357	312.12303	−1.72	C_18_H_18_NO_4_	-	-	-	-	(+): 297
A37	6.87	Protosinomenine	330.17032	330.16998	−1.01	C_19_H_24_NO_4_	-	-	-	-	(+): 192
A38	7.11	Fuzitine	342.16969	342.16998	0.86	C_20_H_24_NO_4_	-	-	-	-	(+): 192
A39	7.13	Oblongine	314.17491	314.17507	0.51	C_19_H_24_NO_3_	-	-	-	-	(+): 269, 237, 175, 143
A40	7.19	Laudanosine	358.20120	358.20128	0.24	C_21_H_28_NO_4_	-	-	-	-	(+): 327, 189
A41	7.29	Deoxyvasicinone	187.08643	187.08659	0.83	C_11_H_11_N_2_O	-	-	-	-	(+): 146
A42	7.35	Unknown	358.20108	358.20128	0.56	C_21_H_28_NO_4_	-	-	-	-	(+): 189
A43	7.43	Menisperine	356.18596	356.18563	−0.92	C_21_H_26_NO_4_^+^	-	-	-	-	(+): 311, 296, 279
A44	7.74	Tetrahydroberberine isomer	340.15482	340.15433	−1.41	C_20_H_22_NO_4_	-	-	-	-	-
A45	7.85	Tembetarine isomer	344.18582	344.18563	−0.54	C_20_H_26_NO_4_^+^	-	-	-	-	(+): 137
A46	7.85	Methylcorydine	356.18579	356.18563	−0.43	C_21_H_26_NO_4_^+^	-	-	-	-	(+): 311, 279
A47	7.93	Pilocarpine isomer	260.12826	260.12812	−0.53	C_15_H_18_NO_3_	-	-	-	-	-
A48	8.09	8-*O*-methyloblongine isomer	328.19121	328.19072	−1.50	C_20_H_26_NO_3_	-	-	-	-	(+): 283
A49	8.27	Tetrahydroberberine isomer	340.15452	340.15433	−0.55	C_20_H_22_NO_4_	-	-	-	-	(+): 309, 192
A50	8.28	1-Methoxyindole-3-acetamide	205.09713	205.09715	0.11	C_11_H_13_N_2_O_2_	-	-	-	-	(+): 188
A51	8.30	Cryptopine	370.16531	370.16490	−1.11	C_21_H_24_NO_5_	-	-	-	-	-
A52	8.49	Isaindigodione isomer	327.13486	327.13393	−2.83	C_18_H_19_N_2_O_4_	-	-	-	-	(+): 201
A53	8.66	5,8,13,13a-tetrahydro-2,9,10,11-tetrahydro-3-methoxy-7-methy-6*H*-dibenz[a,g]quinoline	324.12323	324.12303	−0.60	C_19_H_18_NO_4_	-	-	-	-	(+): 294, 280
A54	8.79	Dasycarpamin	304.15478	304.15433	−1.46	C_17_H_22_NO_4_	-	-	-	-	-
A55	8.85	Armepavine	314.17539	314.17507	−1.00	C_19_H_24_NO_3_	-	-	-	-	(+): 269, 237
A56	9.02	Rotundine	356.18565	356.18563	−0.03	C_21_H_26_NO_4_	-	-	-	-	(+): 192
A57	9.03	Platydesmine	260.12836	260.12812	−0.91	C_15_H_18_NO_3_	-	-	-	-	(+): 242
A58	9.15	Demethyleneberberine	324.12337	324.12303	−1.03	C_19_H_18_NO_4_	-	-	-	-	(+): 309, 308, 294, 280
A59	9.70	Indole-3-acetonitrile-2-*S*-β-D-glucopyranoside	-	-	-	-	349.08596	349.08637	1.17	C_16_H_17_N_2_O_5_S	(−): 187, 160
A60	9.91	Platydesmine isomer	260.12817	260.12812	−0.19	C_15_H_18_NO_3_	-	-	-	-	-
A61	10.00	11-hydroxypalmatine or 13-hydroxypalmatine	368.14971	368.14925	−1.25	C_21_H_22_NO_5_	-	-	-	-	(+): 352, 323, 322
A62	10.00	1-hydroxyberberine	352.11789	352.11795	0.17	C_20_H_18_NO_5_	-	-	-	-	(+): 308
A63	10.10	3-(2′-carboxyphenyl)-4(3H)-quinazolinone	267.07661	267.07642	−0.73	C_15_H_11_N_2_O_3_	-	-	-	-	(+): 249
A64	10.14	*N*-methylcanadine isomer	354.17009	354.16998	−0.28	C_21_H_24_NO_4_	-	-	-	-	(+): 190
A65	10.30	Cryptopine isomer	370.16510	370.16490	−0.55	C_21_H_24_NO_5_	-	-	-	-	-
A66	10.69	Indole-3-acetonitrile-4-methoxy-2-*S-*β-D-glucopyranoside or *N*-Methoxyindole-3-acetonitrile-2-*S*-β-D-glucopyranoside	381.11206	381.11148	−1.51	C_17_H_21_N_2_O_6_S	379.09657	379.09693	0.95	C_17_H_19_N_2_O_6_S	(+): 219
A67	11.10	Berberrubine	322.10757	322.10738	−0.57	C_19_H_16_NO_4_	-	-	-	-	(+): 307
A68	11.10	Tetrahydrocorysamine	338.13873	338.13868	−0.13	C_20_H_20_NO_4_	-	-	-	-	(+): 323, 322, 308, 294
A69	11.11	*N*-methyltetrahydropalmatine	370.20188	370.20128	−1.60	C_22_H_28_NO_4_	-	-	-	-	(+): 206
A70	11.21	Tetrahydroberberine	340.15458	340.15433	−0.73	C_20_H_22_NO_4_	-	-	-	-	(+): 176
A71	11.25	3-(2′-hydroxyphenyl)-4-(3H)-quinazolinone	239.08162	239.08150	−0.50	C_14_H_11_N_2_O_2_	-	-	-	-	(+): 221
A72	11.26	*N*-p-Coumaroyltyramine	284.12834	284.12812	−0.79	C_17_H_18_NO_3_	-	-	-	-	(+): 147
A73	11.30	*N*-methylcanadine	354.17029	354.16998	−0.87	C_21_H_24_NO_4_	-	-	-	-	(+): 190
A74	11.97	Palmatine *	352.15457	352.15433	−0.67	C_21_H_22_NO_4_	-	-	-	-	(+): 336, 308
A75	12.25	Berberine	336.12277	336.12303	0.79	C_20_H_18_NO_4_	-	-	-	-	(+): 320, 292
A76	12.90	9-hydroxy-10-methoxy-13-methyl-5,6-dihydro-[1,3]dioxolo[4,5-g]isoquinolino[3,2-a]isoquinolin-7-ium	336.12296	336.12303	0.22	C_20_H_18_NO_4_	-	-	-	-	(+): 292
A77	14.15	Unknown	486.19116	486.19111	−0.09	C_29_H_28_NO_6_	-	-	-	-	(+): 336
A78	14.38	8-oxopalmatine	368.14933	368.14925	−0.22	C_21_H_22_NO_5_	-	-	-	-	(+): 338
A79	14.72	Canthin-6-one	221.07097	221.07094	−0.13	C_14_H_9_N_2_O	-	-	-	-	(+): 193, 167, 140
A80	15.25	Skimmianine	260.09171	260.09173	0.10	C_14_H_14_NO_4_	-	-	-	-	(+): 227
A81	15.69	γ-fagarine	230.08119	230.08117	−0.07	C_13_H_12_NO_3_	-	-	-	-	(+): 215
A82	17.21	Dictamnine	200.07066	200.07060	−0.30	C_12_H_10_NO_2_	-	-	-	-	(+): 185
A83	18.56	7-hydroxyrutaecarpine	304.10797	304.10805	0.26	C_18_H_14_N_3_O_2_	-	-	-	-	-
A84	19.36	8-oxopalmatine	368.14907	368.14925	0.49	C_21_H_22_NO_5_	-	-	-	-	(+): 338
A85	21.56	Oxyberberine	352.11792	352.11795	0.07	C_20_H_18_NO_5_	-	-	-	-	(+): 337, 322, 294
A86	21.69	Indigo *	263.08146	263.08150	0.18	C_16_H_11_N_2_O_2_	-	-	-	-	(+): 245, 235, 219
A87	22.72	*N*-methylflindersine	242.11759	242.11756	−0.15	C_15_H_16_NO_2_	-	-	-	-	-
A88	23.49	Indirubin *	263.08145	263.08150	0.21	C_16_H_11_N_2_O_2_	-	-	-	-	(+): 245, 235, 219

^a^: [M + HCOO]^−^. -: not detected. * Compared with a reference standard.

**Table 3:** Terpenoids identified in Lanqin oral liquid by UHPLC-FT-ICR MS.

No.	RT (min)	Identification	[M-H]^+^				[M-H]^−^				Product Ion (*m*/*z*)
			Observed	Calculated	Error (ppm)	Formula	Observed	Calculated	Error (ppm)	Formula	
T1	1.75	Gardoside	-	-	-	-	373.11372	373.11402	0.80	C_16_H_21_O_10_	(−): 211, 193, 167, 149, 123
T2	1.96	Aldoxoside	383.13136	383.13125	−0.28 ^a^	C_16_H_24_NaO_9_	405.13976	405.14024	1.17 ^b^	C_17_H_25_O_11_	(−): 359
T3	1.98	Deacetylasperulosidic acid	-	-	-	-	389.10856	389.10894	0.96	C_16_H_21_O_11_	(−): 345, 227, 209, 183, 165
T4	2.05	Shanzhiside	393.13904	393.13914	0.25	C_16_H_25_O_11_	391.12397	391.12459	1.56	C_16_H_23_O_11_	(−): 229, 211, 185, 167, 149
T5	2.34	Geniposidic acid	375.12859	375.12857	−0.04	C_16_H_23_O_10_	373.11374	373.11402	0.76	C_16_H_21_O_10_	(−): 211, 193, 167, 149, 123
T6	2.43	Ixoroside	383.13112	383.13125	0.36 ^a^	C_16_H_24_NaO_9_	405.13997	405.14024	0.65 ^b^	C_17_H_25_O_11_	(−): 359
T7	2.54	6-oxo demethylgenipin 1-*O*-glucopyranoside	-	-	-	-	387.09334	387.09329	−0.14	C_16_H_19_O_11_	(−): 343
T8	2.80	Deacetyl asperulosidic acid methyl ester	427.12108	427.12108	0.00 ^a^	C_17_H_24_NaO_11_	449.12935	449.13006	1.58 ^b^	C_18_H_25_O_13_	(−): 241
T9	3.07	Deacetylasperulosidic acid isomer	391.12351	391.12349	−0.06	C_16_H_23_O_11_	389.10898	389.10894	−0.12	C_16_H_21_O_11_	(−): 345
T10	3.21	Gardenoside	427.12095	427.12108	0.32 ^a^	C_17_H_24_NaO_11_	449.12939	449.13006	1.50 ^b^	C_18_H_25_O_13_	(−): 241
T11	3.49	Jasminoside G	347.17007	347.17004	−0.09	C_16_H_27_O_8_	345.15527	345.15549	0.63	C_16_H_25_O_8_	(−): 165
T12	3.65	Scandoside methyl ester	427.12100	427.12108	0.19 ^a^	C_17_H_24_NaO_11_	449.12977	449.13006	0.66 ^b^	C_18_H_25_O_13_	(−): 241
T13	4.02	Shanzhiside methyl ester isomer	407.15474	407.15479	0.13	C_17_H_27_O_11_	405.13998	405.14024	0.64	C_17_H_25_O_11_	(−): 361, 225
T14	4.53	Jasminoside B isomer	347.16989	347.17004	0.44	C_16_H_27_O_8_	391.16066	391.16097	0.81 ^b^	C_17_H_27_O_10_	(−): 165
T15	4.56	Galioside	427.12080	427.12108	0.66 ^a^	C_17_H_24_NaO_11_	449.13007	449.13006	−0.01 ^b^	C_18_H_25_O_13_	(−): 241
T16	4.66	Loganic acid	-	-	-	-	375.12974	375.12967	−0.19	C_16_H_23_O_10_	(−): 213
T17	4.89	Jasminoside C	329.15945	329.15948	0.09	C_16_H_25_O_7_	327.14492	327.14493	0.01	C_16_H_23_O_7_	(−): 165
T18	4.96	Jasminoside B	347.16974	347.17004	0.89	C_16_H_27_O_8_	391.16044	391.16097	1.36 ^b^	C_17_H_27_O_10_	(−): 345, 179, 165, 161
T19	5.31	Shanzhiside methyl ester	-	-	-	-	405.14012	405.14024	0.29	C_17_H_25_O_11_	(−): 243
T20	5.32	7-epiloganin	-	-	-		389.14535	389.14532	−0.09	C_17_H_25_O_10_	(−): 181, 166
T21	5.65	Mussaenosidic acid	-	-	-	-	375.12956	375.12967	0.29	C_16_H_23_O_10_	(−): 191
T22	5.70	Genipin-1-*O*-β-D-gentiobioside	551.19677	551.19705	0.51	C_23_H_35_O_15_	595.18735	595.18797	1.04 ^b^	C_24_H_35_O_17_	(−): 549, 225, 207, 147, 123, 101
T23	5.72	8-Epiapodantheroside	389.14434	389.14422	−0.31	C_17_H_25_O_10_	387.12895	387.12967	1.86	C_17_H_23_O_10_	-
T24	6.00	Genameside C	551.19796	551.19705	−1.66	C_23_H_35_O_15_	595.18720	595.18797	1.30 ^b^	C_24_H_35_O_17_	(−): 549, 225, 123
T25	6.06	7,8-dihydro-genipin-1-*O*-β-gentiobioside	-	-	-	-	597.20326	597.20362	0.61 ^b^	C_24_H_37_O_17_	(−): 551, 389, 227
T26	6.07	Jasminoside I	493.22826	493.22795	−0.62	C_22_H_37_O_12_	537.21830	537.21888	1.08 ^b^	C_23_H_37_O_14_	(−): 491, 167
T27	6.17	Asperulosidic acid	-	-	-	-	431.11920	431.11950	0.68	C_18_H_23_O_12_	-
T28	6.18	Genipin 1-*O*-apiofuranosyl glucopyranoside	-	-	-	-	565.17704	565.17741	0.65 ^b^	C_23_H_33_O_16_	(−): 519, 225
T29	6.28	Geniposide	411.12648	411.12617	−0.76 ^a^	C_17_H_24_NaO_10_	433.13452	433.13515	1.45 ^b^	C_18_H_25_O_12_	(−): 387, 225, 207, 123, 101
T30	6.39	Genameside D	551.19796	551.19705	−1.66	C_23_H_35_O_15_	595.18773	595.18797	0.41 ^b^	C_24_H_35_O_17_	(−): 549, 387, 225
T31	6.44	Genipin 1-*O*-xylopyranosyl glucopyranoside	-	-	-	-	565.17731	565.17741	0.17 ^b^	C_23_H_33_O_16_	(−): 519, 225, 207
T32	6.60	Dihydrogeniposide	-	-	-	-	435.15050	435.15080	0.68 ^b^	C_18_H_27_O_12_	-
T33	6.69	Jasminoside O	463.21823	463.21739	−1.81	C_21_H_35_O_11_	461.20303	461.20284	−0.42	C_21_H_33_O_11_	(+): 331, 169
T34	6.81	Epijasminoside A or Jasminoside A/E/K	331.17512	331.17513	0.03	C_16_H_27_O_7_	375.16572	375.16606	0.90 ^b^	C_17_H_27_O_9_	(+): 169
T35	6.88	Epijasminoside A or Jasminoside A/E/K	331.17507	331.17513	0.17	C_16_H_27_O_7_	375.16591	375.16606	0.39 ^b^	C_17_H_27_O_9_	(+): 169
T36	7.07	Epijasminoside A or Jasminoside A/E/K	331.17569	331.17513	−1.69	C_16_H_27_O_7_	375.16573	375.16606	0.87 ^b^	C_17_H_27_O_9_	(+): 169
T37	7.34	Jasminoside C	329.15980	329.15948	−0.96	C_16_H_25_O_7_	373.15019	373.15041	0.59 ^b^	C_17_H_25_O_9_	(−): 165
T38	7.36	Genipin	-	-	-	-	225.07668	225.07685	0.75	C_11_H_13_O_5_	(−): 147
T39	7.61	Jasminodiol	-	-	-	-	183.10241	183.10267	1.42	C_10_H_15_O_3_	(−): 139
T40	7.66	6″-*O*-trans-p-coumaroylgenipin gentiobioside isomer	697.23369	697.23383	0.20	C_32_H_41_O_17_	695.21832	695.21927	1.37	C_32_H_39_O_17_	(−): 163
T41	7.83	10-*O*-succinoylgeniposide isomer	-	-	-	-	487.14544	487.14571	0.56	C_21_H_27_O_13_	(−): 207
T42	8.23	(1R,7R,8S,10R)-7,8,11-trihydroxyguai-4-en-3-one-8-*β*-D-glucopyranside	431.22786	431.22756	−0.70	C_21_H_35_O_9_	429.21274	429.21301	0.62	C_21_H_33_O_9_	(+): 269
T43	8.24	10-*O*-acetylgeniposide	453.13736	453.13673	−1.39 ^a^	C_19_H_26_NaO_11_	475.14536	475.14571	0.75 ^b^	C_20_H_27_O_13_	(−): 209
T44	8.37	2′-*O*-trans-p-coumaroylgardoside	-	-	-	-	519.15007	519.15080	1.41	C_25_H_27_O_12_	(−): 163, 145, 123
T45	8.59	6′-*O*-trans-sinapoylgardoside	581.18719	581.18648	−1.21	C_27_H_33_O_14_	579.17142	579.17193	0.87	C_27_H_31_O_14_	(−): 325, 223, 205
T46	8.67	6″-*O*-trans-caffeoylgenipin gentiobioside	-	-	-	-	711.21306	711.21419	1.58	C_32_H_39_O_18_	(−): 179, 123
T47	8.78	6′-*O*-trans-p-coumaroylgeniposidic acid	-	-	-	-	519.15061	519.15080	0.37	C_25_H_27_O_12_	(−): 211, 163, 145, 123
T48	8.88	10-*O*-succinoylgeniposide	-	-	-	-	487.14528	487.14571	0.89	C_21_H_27_O_13_	(−): 207
T49	8.95	10-(6-*O*-trans-sinapoylglucopyranosyl)-gardendiol	567.20828	567.20722	−1.87	C_27_H_35_O_13_	565.19190	565.19266	1.35	C_27_H_33_O_13_	(−): 325, 295, 265, 223
T50	8.99	6′-*O*-trans-sinapoylgardoside isomer	581.18726	581.18648	−1.34	C_27_H_33_O_14_	579.17130	579.17193	1.08	C_27_H_31_O_14_	(−): 325, 223, 205
T51	9.09	Jasminoside R	513.19610	513.19425	−3.61 ^a^	C_22_H_34_NaO_12_	535.20260	535.20323	1.18 ^b^	C_23_H_35_O_14_	-
T52	9.09	6″-*O*-trans-p-coumaroylgenipin gentiobioside isomer	-	-	-	-	695.21812	695.21927	1.66	C_32_H_39_O_17_	(−): 469, 163
T53	9.31	11-(6-*O*-trans-sinapoylglucopyranosyl)-gardendiol	567.20835	567.20722	−1.99	C_27_H_35_O_13_	565.19236	565.19266	0.55	C_27_H_33_O_13_	(−): 265, 223
T54	9.41	10-*O*-acetylgeniposide isomer	453.13754	453.13673	−1.78 ^a^	C_19_H_26_NaO_11_	475.14530	475.14571	0.87 ^b^	C_20_H_27_O_13_	(−): 209
T55	9.72	6″-*O*-trans-p-coumaroylgenipin gentiobioside	697.23461	697.23383	−1.12	C_32_H_41_O_17_	695.21845	695.21927	1.18	C_32_H_39_O_17_	(−): 469, 367, 349, 307, 265, 235, 207, 163, 145, 123
T56	9.75	Jasminoside I/S/H	-	-	-	-	537.21830	537.21888	1.08 ^b^	C_23_H_37_O_14_	(−): 491
T57	9.91	6″-*O*-trans-sinapoylgenipin gentiobioside	-	-	-	-	755.23881	755.24040	2.11	C_34_H_43_O_19_	(−): 427, 325, 265, 223, 205
T58	10.05	6″-*O*-trans-feruloylgenipin gentiobioside	727.24630	727.24439	−2.63	C_33_H_43_O_18_	725.22867	725.22984	1.61	C_33_H_41_O_18_	(−): 397, 295, 235, 225, 207, 193, 175, 123
T59	10.33	Jasminoside R	-	-	-	-	489.19730	489.19775	0.92	C_22_H_33_O_12_	-
T60	10.34	Crocin I	-	-	-	-	975.37046	975.37148	1.04	C_44_H_63_O_24_	(−): 651
T61	10.68	6′-trans-sinapoylgeniposide isomer	617.18520	617.18408	−1.82 ^a^	C_28_H_34_NaO_14_	593.18700	593.18758	0.97	C_28_H_33_O_14_	(−): 367, 223
T62	10.75	6′-*O*-trans-sinapoyljasminoside L	553.22916	553.22795	−2.19	C_27_H_37_O_12_	551.21267	551.21340	1.32	C_27_H_35_O_12_	(−): 223
T63	11.36	6′-trans-sinapoylgeniposide	595.20406	595.20213	−3.24	C_28_H_35_O_14_	593.18666	593.18758	1.55	C_28_H_33_O_14_	(−): 367, 223, 205, 123
T64	11.47	6′-*O*-trans-coumaroylgeniposide	535.18261	535.18100	−3.00	C_26_H_31_O_12_	533.16592	533.16645	1.00	C_26_H_29_O_12_	(−): 307, 145
T65	11.70	Jasminoside I/S/H	-	-	-	-	491.21363	491.21340	−0.47	C_22_H_35_O_12_	-
T66	11.72	6″-*O*-trans-p-coumaroylgenipin gentiobioside isomer	-	-	-	-	695.21805	695.21927	1.75	C_32_H_39_O_17_	(−): 207, 163
T67	12.06	Jasminoside I/S/H	-	-	-	-	491.21248	491.21340	1.87	C_22_H_35_O_12_	-
T68	12.10	Rehmapicrogenin or isomer	-	-	-	-	183.10239	183.10267	1.50	C_10_H_15_O_3_	-
T69	12.42	Rutaevin	487.19808	487.19626	−3.74	C_26_H_31_O_9_	485.18117	485.18171	1.10	C_26_H_29_O_9_	(−): 467, 423
T70	13.27	6″-*O*-trans-p-cinnamoylgenipin gentiobioside	703.22355	703.22086	−3.83 ^a^	C_32_H_40_NaO_16_	725.22811	725.22984	2.38 ^b^	C_33_H_41_O_18_	(−): 679, 531, 355, 225, 207, 147, 123
T71	13.97	6′-*O*-trans-sinapoyljasminoside A	537.23366	537.23304	−1.15	C_27_H_37_O_11_	535.21809	535.21849	0.74	C_27_H_35_O_11_	(−): 223
T72	14.44	Obacunoic acid	473.21640	473.21699	1.26	C_26_H_33_O_8_	471.20217	471.20244	0.59	C_26_H_31_O_8_	(−): 325
T73	14.47	Crocin I acid	-	-	-	-	1007.37486	1007.37656	1.69	C_48_H_63_O_23_	(−): 683
T74	14.63	6′-trans-sinapoylgeniposide isomer	617.18462	617.18408	−0.89 ^a^	C_28_H_34_NaO_14_	593.18691	593.18758	1.14	C_28_H_33_O_14_	(−): 367, 223, 205, 123
T75	14.75	6′-*O*-trans-sinapoyjasminoside C	535.21774	535.21739	−0.65	C_27_H_35_O_11_	533.20202	533.20284	1.53	C_27_H_33_O_11_	(−): 223, 205
T76	15.40	12α-hydroxylimonin	487.19607	487.19626	0.38	C_26_H_31_O_9_	-	-	-	-	-
T77	16.49	Neocrocin E	-	-	-	-	975.37036	975.37148	1.15	C_44_H_63_O_24_	(−): 651
T78	16.86	Crocin-Ⅲ or 13-cis-crocetin-8′-*O*-β-D-gentiobioside or isomer	653.28032	653.28038	0.09	C_32_H_45_O_14_	651.26523	651.26583	0.92	C_32_H_43_O_14_	(−): 327, 283, 239
T79	17.91	Crocin II	837.31452	837.31515	0.75 ^a^	C_38_H_54_NaO_19_	813.31723	813.31865	1.75	C_38_H_53_O_19_	(−): 651
T80	17.93	beta-D-glucosyl crocetin	491.22716	491.22756	0.82	C_26_H_35_O_9_	489.21276	489.21301	0.50	C_26_H_33_O_9_	(−): 327
T81	18.35	6′-*O*-trans-sinapoyljasminoside A	-	-	-	-	535.21815	535.21849	0.62	C_27_H_35_O_11_	(−): 223
T82	18.37	beta-D-glucosyl crocetin	491.22719	491.22756	0.76	C_26_H_35_O_9_	489.21277	489.21301	0.48	C_26_H_33_O_9_	(−): 327
T83	19.14	6′-*O*-trans-sinapoyljasminoside A isomer	-	-	-	-	535.21823	535.21849	0.49	C_27_H_35_O_11_	(−): 223
T84	19.60	Obaculactone	471.20104	471.20134	0.64	C_26_H_31_O_8_	469.18655	469.18679	0.51	C_26_H_29_O_8_	(+): 453, 427, 425, 409, 367, 339, 161
T85	20.34	Crocin II	837.31579	837.31515	−0.77 ^a^	C_38_H_54_NaO_19_	813.31960	813.31865	−1.17	C_38_H_53_O_19_	(−): 651
T86	20.35	Obacunoic acid	473.21675	473.21699	0.52	C_26_H_33_O_8_	471.20245	471.20244	−0.02	C_26_H_31_O_8_	(−): 325, 307
T87	20.70	Crocin-Ⅲ or 13-cis-crocetin-8′-*O*-β-D-gentiobioside or isomer	675.26198	675.26233	0.52 ^a^	C_32_H_44_NaO_14_	651.26503	651.26583	1.22	C_32_H_43_O_14_	(−): 327, 283, 239
T88	21.09	Obacunoic acid isomer	473.21690	473.21699	0.20	C_26_H_33_O_8_	471.20262	471.20244	−0.39	C_26_H_31_O_8_	(−): 325
T89	21.19	Neocrocin B	-	-	-	-	987.35048	987.35035	−0.14	C_48_H_59_O_22_	-
T90	21.38	Crocin-Ⅲ or 13-cis-crocetin-8′-*O*-β-D-gentiobioside or isomer	653.28052	653.28038	−0.21	C_32_H_45_O_14_	651.26515	651.26583	1.04	C_32_H_43_O_14_	(−): 327
T91	21.95	Dehydrolimonin	469.18526	469.18569	0.93	C_26_H_29_O_8_	513.17635	513.17662	0.53 ^b^	C_27_H_29_O_10_	(−): 467, 449
T92	22.05	Crocin-Ⅲ or 13-cis-crocetin-8′-*O*-β-D-gentiobioside or isomer	653.27972	653.28038	1.02	C_32_H_45_O_14_	651.26462	651.26583	1.86	C_32_H_43_O_14_	(−): 327
T93	22.90	beta-D-glucosylcrocetin	491.22735	491.22756	0.43	C_26_H_35_O_9_	489.21272	489.21301	0.58	C_26_H_33_O_9_	(−): 327
T94	23.14	beta-D-glucosylcrocetin	491.22726	491.22756	0.61	C_26_H_35_O_9_	489.21268	489.21301	0.68	C_26_H_33_O_9_	(−): 327
T95	23.47	beta-D-glucosylcrocetin	491.22722	491.22756	0.69	C_26_H_35_O_9_	489.21265	489.21301	0.72	C_26_H_33_O_9_	(−): 327
T96	24.02	Obacunone	455.20596	455.20643	1.04	C_26_H_31_O_7_	453.19169	453.19188	0.40	C_26_H_29_O_7_	(+): 437, 419, 409, 391, 359, 349, 331, 315
T97	25.09	Crocetin	329.17453	329.17474	0.63	C_20_H_25_O_4_	327.15993	327.16018	0.77	C_20_H_23_O_4_	(−): 283
T98	25.36	Erubigenin	489.35728	489.35745	0.35	C_30_H_49_O_5_	487.34244	487.34290	0.93	C_30_H_47_O_5_	(−): 469
T99	25.68	Dikamaliartanes A	501.32111	501.32107	−0.08	C_30_H_45_O_6_	499.30640	499.30651	0.23	C_30_H_43_O_6_	-
T100	25.83	Crocetin isomer	329.17472	329.17474	0.04	C_20_H_25_O_4_	327.15992	327.16018	0.81	C_20_H_23_O_4_	(−): 283
T101	25.95	Obacunone isomer	455.20628	455.20643	0.33	C_26_H_31_O_7_	453.19150	453.19188	0.83	C_26_H_29_O_7_	(+): 437, 411, 409, 359
T102	25.96	Gardenic acid B	487.34171	487.34180	0.19	C_30_H_47_O_5_	485.32690	485.32725	0.71	C_30_H_45_O_5_	-
T103	26.56	Crocetin isomer	329.17474	329.17474	0.00	C_20_H_25_O_4_	327.15986	327.16018	0.99	C_20_H_23_O_4_	(−): 283
T104	27.17	Crocetin isomer	329.17470	329.17474	0.10	C_20_H_25_O_4_	327.15981	327.16018	1.14	C_20_H_23_O_4_	(−): 283
T105	27.78	27-*O*-p-(E)-coumaroyloxyursolic acid	-	-	-	-	617.38369	617.38476	1.73	C_39_H_54_O_6_	-
T106	29.02	9,19-cyclolanost-24-ene-3,23-dione	-	-	-	-	469.33097	469.33233	2.91	C_30_H_45_O_4_	-
T107	29.52	Quadrangularic acid E	-	-	-	-	471.34652	471.34798	3.10	C_30_H_47_O_4_	-
T108	30.44	Betulinic acid	-	-	-	-	455.35162	455.35307	3.19	C_30_H_47_O_3_	(−): 407

^a^: [M + Na]^+^; ^b^: [M + HCOO]^−^. -: not detected.

**Table 4:** Organic acids identified in Lanqin oral liquid by UHPLC-FT-ICR MS.

No.	RT (min)	Identification	[M-H]^+^				[M-H]^−^				Product Ion (*m*/*z*)
			Observed	Calculated	Error (ppm)	Formula	Observed	Calculated	Error (ppm)	Formula	
O1	0.94	Quinic acid	-	-	-	-	191.05582	191.05611	1.55	C_7_H_11_O_6_	(−): 173, 127
O2	1.27	Succinic acid	-	-	-	-	117.01917	117.01933	1.37	C_4_H_5_O_4_	-
O3	1.32	Malic acid	-	-	-	-	133.01404	133.01425	1.59	C_4_H_5_O_5_	(−): 115
O4	1.69	Quinic acid 3-hydroxy-3-methylpentanedioate	337.11289	337.11292	0.10	C_13_H_21_O_10_	335.09793	335.09837	1.31	C_13_H_19_O_10_	(−): 191
O5	2.19	Dihydroxybenzoic acid glucoside	-	-	-	-	315.07186	315.07216	0.93	C_13_H_15_O_9_	(−): 153, 109
O6	2.27	Vanillic acid hexose	331.10239	331.10236	−0.09	C_14_H_19_O_9_	329.08737	329.08781	1.32	C_14_H_17_O_9_	(−): 167
O7	2.51	3-({3-[4-(β-glucopyranosyloxy)-3-methoxyphenyl]-2-propenoyl}oxy)-1,4,5-trihydroxycyclohexanecarboxylic acid	531.17052	531.17083	0.58	C_23_H_31_O_14_	529.15607	529.15628	0.40	C_23_H_29_O_14_	(−): 193, 191
O8	2.59	Dihydroxybenzoic acid glucoside isomer	317.08701	317.08671	−0.95	C_13_H_17_O_9_	315.07189	315.07216	0.85	C_13_H_15_O_9_	(−): 153, 109
O9	3.41	Neochlorogenic acid	355.10206	355.10236	0.83	C_16_H_19_O_9_	353.08749	353.08781	0.91	C_16_H_17_O_9_	(−): 191, 179, 173, 135
O10	3.81	4-*O*-caffeoyl-3-*O*-feruloylquinic acid	531.17059	531.17083	0.45	C_23_H_31_O_14_	529.15610	529.15628	0.34	C_23_H_29_O_14_	(−): 191, 179, 173
O11	5.19	4-*O*-caffeoyl-3-*O*-feruloylquinic acid isomer	531.17049	531.17083	0.64	C_23_H_31_O_14_	529.15606	529.15628	0.41	C_23_H_29_O_14_	(−): 191, 179, 173
O12	5.27	3-p-coumaroylquinic acid	339.10747	339.10744	−0.08	C_16_H_19_O_8_	337.09277	337.09289	0.35	C_16_H_17_O_8_	(−): 191, 163
O13	5.60	Chlorogenic acid *	355.10232	355.10236	0.11	C_16_H_19_O_9_	353.08757	353.08781	0.67	C_16_H_17_O_9_	(−): 191, 179, 173, 135
O14	5.83	5-*O*-feruloylquinic acid	369.11802	369.11801	−0.02	C_17_H_21_O_9_	367.10306	367.10346	1.08	C_17_H_19_O_9_	(−): 193, 191, 173, 149, 134
O15	5.85	Cryptochlorogenic acid	355.10257	355.10236	−0.59	C_16_H_19_O_9_	353.08759	353.08781	0.62	C_16_H_17_O_9_	(−): 191, 179, 173, 135
O16	6.04	4-sinapoylquinic acid	-	-	-	-	397.11376	397.11402	0.65	C_18_H_21_O_10_	(−): 223, 191, 169, 146
O17	6.17	Caffeic acid *	-	-	-	-	179.03487	179.03498	0.64	C_9_H_7_O_4_	(−): 135
O18	6.58	4-p-coumaroylquinic acid	339.10753	339.10744	−0.25	C_16_H_19_O_8_	337.09281	337.09289	0.26	C_16_H_17_O_8_	(−): 191, 163
O19	6.60	3,4-dicaffeoyl quinic acid isomer	517.13521	517.13405	−2.24	C_25_H_25_O_12_	515.11895	515.11950	1.07	C_25_H_23_O_12_	(−): 353, 191, 179, 135
O20	6.62	4-*O*-β-D-glucosyl-cis-*p*-coumaric acid	-	-	-	-	325.09285	325.09289	0.13	C_15_H_17_O_8_	-
O21	6.67	2-hydroxy-1,4-phthalic acid	-	-	-	-	181.01400	181.01425	1.39	C_8_H_5_O_5_	(−): 137
O22	6.92	3-*O*-feruloylquinic acid	369.11805	369.11801	−0.12	C_17_H_21_O_9_	367.10342	367.10346	0.10	C_17_H_19_O_9_	(−): 193, 191, 173, 134
O23	7.06	3-Sinapoylquinic acid	399.12895	399.12857	−0.94	C_18_H_23_O_10_	397.11398	397.11402	0.11	C_18_H_21_O_10_	(−): 223, 191, 169, 146
O24	7.23	Dicaffeoyl quinic acid-glucopyranoside	-	-	-	-	677.17142	677.17232	1.33	C_31_H_33_O_17_	-
O25	7.57	4-*O*-feruloylquinic acid	369.11813	369.11801	−0.34	C_17_H_21_O_9_	367.10337	367.10346	0.24	C_17_H_19_O_9_	(−): 193, 191, 173
O26	7.75	Dicaffeoyl quinic acid-glucopyranoside	-	-	-	-	677.17156	677.17232	1.13	C_31_H_33_O_17_	(−): 515
O27	8.05	Dicaffeoyl quinic acid-glucopyranoside	-	-	-	-	677.17136	677.17232	1.42	C_31_H_33_O_17_	(−): 515
O28	8.14	Ferulic acid	195.06547	195.06519	−1.46	C_10_H_11_O_4_	193.05029	193.05063	1.79	C_10_H_9_O_4_	(−): 149
O29	8.42	Ferulic acid isomer	-	-	-	-	193.05041	193.05063	1.16	C_10_H_9_O_4_	(−): 149
O30	8.73	3,4-dicaffeoyl quinic acid	517.13626	517.13405	−4.27	C_25_H_25_O_12_	515.11906	515.11950	0.86	C_25_H_23_O_12_	(−): 353, 191, 179, 135
O31	9.13	3,5-dicaffeoyl quinic acid	517.13648	517.13405	−4.69	C_25_H_25_O_12_	515.11893	515.11950	1.11	C_25_H_23_O_12_	(−): 353, 191, 179, 135
O32	9.65	4,5-dicaffeoyl quinic acid	517.13521	517.13405	−2.24	C_25_H_25_O_12_	515.11877	515.11950	1.42	C_25_H_23_O_12_	(−): 353, 191, 179, 135
O33	9.99	3-*O*-caffeoyl-4-*O*-sinapoylquinic acid	561.16097	561.16027	−1.26	C_27_H_29_O_13_	559.14509	559.14571	1.12	C_27_H_27_O_13_	(−): 379, 364, 353, 335, 223, 205, 191, 179, 173, 164, 161, 155, 149, 137, 135
O34	10.02	4-p-coumaroylquinic acid isomer	339.10758	339.10744	−0.39	C_16_H_19_O_8_	-	-	-	-	-
O35	10.17	3,5-di-*O*-caffeoyl-4-*O*-(3-hydroxy-3-methyl) glutarylquinic acid	661.17765	661.17631	−2.02	C_31_H_33_O_16_	659.16151	659.16176	0.38	C_31_H_31_O_16_	(−): 353, 191
O36	10.82	5-*O*-caffeoyl-4-*O*-sinapoylquinic acid	561.16144	561.16027	−2.09	C_27_H_29_O_13_	559.14515	559.14571	1.00	C_27_H_27_O_13_	(−): 379, 364, 353, 335, 223, 205, 191, 179, 173, 164, 161, 155, 149, 137, 135
O37	11.17	5-*O*-caffeoyl-4-*O*-sinapoylquinic acid isomer	561.16144	561.16027	−2.09	C_27_H_29_O_13_	559.14501	559.14571	1.26	C_27_H_27_O_13_	(−): 379, 364, 353, 335, 223, 205, 191, 179, 173, 164, 161, 155, 149, 137, 135
O38	12.55	Phellinsin A or isomer	359.07730	359.07614	−3.22	C_18_H_15_O_8_	357.06125	357.06159	0.96	C_18_H_13_O_8_	(−): 339, 321, 313, 295
O39	15.59	9,12-dihydroxy-13-oxooctadec-14-enoic acid	329.23207	329.23225	0.55	C_18_H_33_O_5_	327.21739	327.21770	0.94	C_18_H_31_O_5_	(−): 291, 273, 247, 239, 229, 211, 209, 203, 197, 193, 183, 171
O40	15.87	9,12-dihydroxy-13-oxooctadec-14-enoic acid isomer	329.23259	329.23225	−1.03	C_18_H_33_O_5_	327.21725	327.21770	1.37	C_18_H_31_O_5_	(−): 291, 273, 247, 239, 229, 211, 209, 203, 197, 193, 183, 171
O41	17.04	9,12-dihydroxy-13-oxooctadecanoic acid	353.22966	353.22984	0.51 ^a^	C_18_H_34_NaO_5_	329.23302	329.23335	1.01	C_18_H_33_O_5_	(−): 311, 229, 211, 183, 171
O42	18.71	9,12-dihydroxy-13-oxooctadecanoic acid isomer	353.22964	353.22984	0.59 ^a^	C_18_H_34_NaO_5_	329.23322	329.23335	0.40	C_18_H_33_O_5_	(−): 311, 229, 211
O43	18.94	9,12-dihydroxy-13-oxooctadecanoic acid isomer	353.22958	353.22984	0.75 ^a^	C_18_H_34_NaO_5_	329.23317	329.23335	0.54	C_18_H_33_O_5_	(−): 311, 229, 211
O44	19.64	9,12-dihydroxy-13-oxooctadecanoic acid isomer	353.22977	353.22984	0.20 ^a^	C_18_H_34_NaO_5_	329.23308	329.23335	0.80	C_18_H_33_O_5_	(−): 311, 229, 211
O45	20.54	9,12-dihydroxy-13-oxooctadecanoic acid isomer	353.22972	353.22984	0.37 ^a^	C_18_H_34_NaO_5_	329.23337	329.23335	−0.08	C_18_H_33_O_5_	(−): 311, 229, 211
O46	23.27	(9Z,12Z)-15,16-dihydroxyoctadeca-9,12-dienoic acid	335.21901	335.21928	0.80 ^a^	C_18_H_32_NaO_4_	311.22236	311.22278	1.36	C_18_H_31_O_4_	(−): 293, 275, 223, 205
O47	25.00	9/12-Octadecanedioic acid or isomer	337.23464	337.23493	0.87 ^a^	C_18_H_34_NaO_4_	313.23799	313.23843	1.42	C_18_H_33_O_4_	(−): 295, 277
O48	25.30	9/12-Octadecanedioic acid or isomer	337.23471	337.23493	0.65 ^a^	C_18_H_34_NaO_4_	313.23821	313.23843	0.71	C_18_H_33_O_4_	(−): 295
O49	29.94	γ-Linolenic acid	-	-	-	-	277.21655	277.21730	2.73	C_18_H_29_O_2_	(−): 233
O50	30.69	Linoleic acid	-	-	-	-	279.23222	279.23295	2.62	C_18_H_31_O_2_	(−): 261
O51	31.59	Oleic acid	283.26315	283.26316	0.02	C_18_H_35_O_2_	281.24796	281.24860	2.30	C_18_H_33_O_2_	-

^a^: [M + Na]^+^. -: not detected. * Compared with a reference standard.

**Table 5:** Phenylpropanoids and other compounds identified in Lanqin oral liquid by UHPLC-FT-ICR MS.

No.	RT (min)	Identification	[M-H]^+^				[M-H]^−^				Product Ion (*m*/*z*)
			Observed	Calculated	Error (ppm)	Formula	Observed	Calculated	Error (ppm)	Formula	
OC1	0.90	Arginine	175.11906	175.11895	−0.61	C_6_H_15_N_4_O_2_	173.10429	173.10440	0.63	C_6_H_13_N_4_O_2_	(+): 158
OC2	1.16	Cytidine	244.09273	244.09280	0.28	C_9_H_14_N_3_O_5_	-	-	-	-	(+): 112
OC3	1.37	Tyrosine	182.08116	182.08117	0.07	C_9_H_12_NO_3_	-	-	-	-	(+): 165, 147, 136
OC4	1.37	Uridine	245.07684	245.07681	−0.11	C_9_H_13_N_2_O_6_	243.06197	243.06226	1.21	C_9_H_11_N_2_O_6_	(−): 110
OC5	1.39	Adenosine	268.10389	268.10403	0.53	C_10_H_14_N_5_O_4_	-	-	-	-	(+): 136
OC6	1.41	Guanosine	284.09891	284.09894	0.11	C_10_H_14_N_5_O_5_	282.08390	282.08439	1.74	C_10_H_12_N_5_O_5_	(−): 150, 133
OC7	1.60	Unknown	363.16472	363.16496	0.66	C_16_H_27_O_9_	361.15007	361.15041	0.94	C_16_H_25_O_9_	(−): 181, 137
OC8	2.05	Phenylalanine hexose	328.13890	328.13908	0.55	C_15_H_22_NO_7_	326.12431	326.12453	0.66	C_15_H_20_NO_7_	(−): 164
OC9	2.11	Phenylalanine	166.08639	166.08626	−0.82	C_9_H_12_NO_2_	-	-	-	-	(+): 149
OC10	2.36	Unknown	363.16535	363.16496	−1.08	C_16_H_27_O_9_	361.15003	361.15041	1.05	C_16_H_25_O_9_	(−): 181, 137
OC11	2.82	4-(2-hydroxyethyl)phenyl 6-*O*-apiofuranosyl-β-D-glucopyranoside	433.17064	433.17044	−0.47	C_19_H_29_O_11_	477.16109	477.16136	0.57 ^c^	C_20_H_29_O_13_	(−): 431, 293
OC12	2.88	1-*O*-syringoyl-β-D-glucopyranoside	361.11286	361.11292	0.18	C_15_H_21_O_10_	359.09821	359.09837	0.43	C_15_H_19_O_10_	(−): 197
OC13	3.81	Tryptophan	205.09704	205.09715	0.57	C_11_H_13_N_2_O_2_	203.08247	203.08260	0.67	C_11_H_11_N_2_O_2_	(+): 188, 146
P1	4.40	Esculin	341.08642	341.08671	0.85	C_15_H_17_O_9_	339.07211	339.07216	0.13	C_15_H_15_O_9_	(+): 179, 151, 133, 123
OC14	4.80	Osmanthuside H	-	-	-	-	431.15579	431.15589	0.23	C_19_H_27_O_11_	(−): 191, 149
P2	5.03	Coniferin	365.12059	365.12069	0.27 ^a^	C_16_H_22_NaO_8_	387.12932	387.12967	0.90 ^c^	C_17_H_23_O_10_	(−): 341, 179
OC15	5.13	Darendoside A	433.17064	433.17044	−0.47	C_19_H_29_O_11_	431.15566	431.15589	0.52	C_19_H_27_O_11_	(−): 299, 191, 149
P3	5.18	Liriodendrin isomer	-	-	-	-	787.26642	787.26662	0.25 ^c^	C_35_H_47_O_20_	(−): 741
OC16	5.39	Darendoside A isomer	433.17153	433.17044	−2.52	C_19_H_29_O_11_	431.15558	431.15589	0.71	C_19_H_27_O_11_	(−): 299, 191, 149
P4	5.61	Syringin	390.17599	390.17586	−0.34 ^b^	C_17_H_28_NO_9_	417.13995	417.14024	0.68 ^c^	C_18_H_25_O_11_	(−): 209
P5	6.09	Esculetin *	-	-	-	-	177.01901	177.01933	1.82	C_9_H_5_O_4_	(−): 149, 133
P6	6.18	Clemastanin B	-	-	-	-	729.26007	729.26114	1.46 ^c^	C_33_H_45_O_18_	(−): 683, 521, 359, 329
OC17	6.41	Unknown	-	-	-	-	563.16146	563.16176	0.52 ^c^	C_23_H_31_O_16_	(−): 517, 225
P7	6.42	Lariciresinol-4′-*O*-β-D-glucoside	-	-	-	-	521.20303	521.20284	−0.37	C_26_H_33_O_11_	(−): 359
OC18	6.59	Sinapyglucoside	387.12897	387.12857	−1.03	C_17_H_23_O_10_	385.11395	385.11402	0.17	C_17_H_21_O_10_	(−): 205
OC19	7.02	2-methyl-L-erythritol-4-*O*-(6-*O*-transsinapoyl)-β-D-glucopyranoside	527.17422	527.17351	−1.34 ^a^	C_22_H_32_NaO_13_	503.17659	503.17701	0.85	C_22_H_31_O_13_	(−): 205
P8	7.09	Liriodendrin	-	-	-	-	787.26595	787.26662	0.85 ^c^	C_35_H_47_O_20_	(−): 741
P9	7.27	5,5′-dimethoxy lariciresinol-4′-*O*-glucoside isomer	605.22181	605.22046	−2.22 ^a^	C_28_H_38_NaO_13_	581.22325	581.22396	1.23	C_28_H_37_O_13_	(−): 415
P10	7.40	(−)-Secoisolariciresinol 4-*O*-β-D-glucopyranoside	-	-	-	-	523.21827	523.21849	0.41	C_26_H_35_O_11_	(−): 361
OC20	7.43	Darendroside B	-	-	-	-	475.18169	475.18210	0.87	C_21_H_31_O_12_	(−): 329
OC21	7.53	1-*O*-feruloyl heptopyranose	409.11089	409.11052	−0.91 ^a^	C_17_H_22_NaO_10_	385.11362	385.11402	1.05	C_17_H_21_O_10_	(+): 387
P11	7.97	3,3′,4-trihydroxy-4-methoxy-7,7-epoxylignan or isomer	331.15427	331.15400	−0.82	C_19_H_23_O_5_	329.13885	329.13945	1.81	C_19_H_21_O_5_	(+): 316
P12	7.97	Lariciresinol-4-*O*-β-D-glucoside	-	-	-	-	521.20218	521.20284	1.27	C_26_H_33_O_11_	(−): 359
P13	8.06	5,5′-dimethoxy lariciresinol-4′-*O*-glucoside	-	-	-	-	581.22346	581.22396	0.87	C_28_H_37_O_13_	(−): 419
P14	8.15	Staunoside C	-	-	-	-	595.20287	595.20323	0.60	C_28_H_35_O_14_	-
P15	8.16	Prinsepiol	-	-	-	-	389.12383	389.12419	0.92	C_20_H_21_O_8_	(−): 374, 359, 175, 151, 136
OC22	8.22	Acteoside isomer	647.19555	647.19464	−1.40 ^a^	C_29_H_36_NaO_15_	623.19736	623.19814	1.25	C_29_H_35_O_15_	(−): 461, 315, 179, 161
P16	8.23	Conicaoside	575.21087	575.20990	−1.69 ^a^	C_27_H_36_NaO_12_	551.21302	551.21340	0.70	C_27_H_35_O_12_	-
OC23	8.82	Acteoside	-	-	-	-	623.19721	623.19814	1.49	C_29_H_35_O_15_	(−): 461, 315, 179, 161
P17	8.87	Isolariciresinol	361.16492	361.16456	−0.98	C_20_H_25_O_6_	405.15502	405.15549	1.16 ^c^	C_21_H_25_O_8_	(−): 359
P18	8.91	Pinoresinol-4-*O*-glucoside	-	-	-	-	519.18674	519.18719	0.86	C_26_H_31_O_11_	(−): 357, 342, 151, 136
P19	9.08	Aschantin	401.16042	401.15948	−2.34	C_22_H_25_O_7_	-	-	-	-	(−): 383
P20	9.12	Acanthoside B	603.20697	603.20481	−3.58 ^a^	C_28_H_36_NaO_13_	579.20790	579.20831	0.72	C_28_H_35_O_13_	(−): 417, 402, 387, 181, 166
P21	10.30	8-hydroxypinoresinol	375.14347	375.14383	0.95	C_20_H_23_O_7_	373.12925	373.12928	0.07	C_20_H_21_O_7_	(−): 313
P22	10.38	8-hydroxysyringaresinol	435.16542	435.16496	−1.07	C_22_H_27_O_9_	433.15003	433.15041	0.87	C_22_H_25_O_9_	(−): 403, 385, 373, 358, 343, 325
P23	10.38	Pinoresinol-4-*O*-glucoside isomer	-	-	-	-	519.18688	519.18719	0.58	C_26_H_31_O_11_	(−): 357
P24	10.41	Secoisolariciresinol	-	-	-	-	361.16543	361.16566	0.65	C_20_H_25_O_6_	(−): 346, 165
P25	10.47	Hydroxymedioresinol	-	-	-	-	403.13921	403.13984	1.58	C_21_H_23_O_8_	(−): 343
OC24	10.52	Leucosceptoside A or isomer	639.22994	639.22835	−2.49	C_30_H_39_O_15_	637.21278	637.21379	1.60	C_30_H_37_O_15_	(−): 461
F40	10.60	Syringetin	347.07655	347.07614	−1.17	C_17_H_15_O_8_	345.06107	345.06159	1.50	C_17_H_13_O_8_	(+): 332
P26	10.93	Lariciresinol-9-*O*-β-D-glucopyranoside	523.21844	523.21739	−2.00	C_26_H_35_O_11_	521.20242	521.20284	0.80	C_26_H_33_O_11_	(−): 506
P27	11.24	3,3′,4-trihydroxy-4-methoxy-7,7-epoxylignan or isomer	331.15428	331.15400	−0.85	C_19_H_23_O_5_	329.13936	329.13945	0.27	C_19_H_21_O_5_	-
P28	11.26	Hydroxymedioresinol isomer	-	-	-	-	403.13953	403.13984	0.77	C_21_H_23_O_8_	-
OC25	11.58	Cistanoside D	-	-	-	-	651.22826	651.22944	1.81	C_31_H_39_O_15_	(−): 505
OC26	12.49	Isomartynoside	-	-	-	-	651.22861	651.22944	1.27	C_31_H_39_O_15_	(−): 505
OC27	13.28	Ethyl caffeate	-	-	-	-	207.06606	207.06628	1.08	C_11_H_11_O_4_	(−): 179, 161, 135
OC28	13.49	Variolaric acid	315.05025	315.04993	−1.03	C_16_H_11_O_7_	313.03517	313.03538	0.66	C_16_H_9_O_7_	(−): 269
P29	14.09	Unknown	329.06581	329.06558	−0.71	C_17_H_13_O_7_	327.05071	327.05103	0.95	C_17_H_11_O_7_	(−): 299, 283, 265, 255, 237, 222, 209
OC29	16.83	Lariciresinol	361.16492	361.16456	−0.98	C_20_H_25_O_6_	359.14979	359.15001	0.61	C_20_H_23_O_6_	(−): 329
OC30	17.58	Unknown	729.14456	729.14501	0.62	C_37_H_29_O_16_	727.12966	727.13046	1.10	C_37_H_27_O_16_	(+): 553, 283
OC31	19.81	Unknown	729.14475	729.14501	0.35	C_37_H_29_O_16_	727.12995	727.13046	0.70	C_37_H_27_O_16_	(+): 553, 283

^a^: [M + Na]^+^; ^b^: [M + NH_4_]^+^; ^c^: [M + HCOO]^−^. -: not detected. * Compared with a reference standard. P: phenylpropanoid; OC: other compound.

## Data Availability

Data are contained within the article.
